# A 5′-BODIPY End-label for Monitoring DNA Duplex-Quadruplex Exchange

**DOI:** 10.1038/s41598-018-35352-0

**Published:** 2018-11-15

**Authors:** Prashant S. Deore, Dmitriy V. Soldatov, Richard A. Manderville

**Affiliations:** Departments of Chemistry and Toxicology, University of Guelph, Guelph, Ontario N1G 2W1 Canada

## Abstract

Fluorescent probes that can distinguish different DNA topologies through changes in optical readout are sought after for DNA-based diagnostics. In this work, the 4,4-difluoro-4-bora-3a, 4a-diaza-s-indacene (BODIPY) chromophore attached to cyanophenyl substituents (BODIPY-CN) has been tethered to the 5′-end of the 15-mer thrombin binding aptamer (TBA) that contains the guanine (G) nucleobase. TBA folds into a unimolecular antiparallel G-quadruplex (GQ) upon binding thrombin and certain metal ions. The 5′-BODIPY-CN-TBA sample possesses a Stokes shift of ~40 nm with wavelengths of excitation/emission at 550/590 nm and exhibits a 2-fold increase in emission intensity compared to the free BODIPY-CN in aqueous buffer that possesses a brightness (εΦ_*fl*_) of ~16,956 M^−1^. cm^−1^. However, when 5′-BODIPY-CN-TBA is base-paired to a complementary strand in the B-form duplex, the emission of the BODIPY-CN end-label increases 7-fold, 14-fold compared to the free-dye. This signal-on response enables the BODIPY-CN end-label to serve as a quencher-free fluorescent probe for monitoring duplex-GQ exchange. The visible end-label minimally perturbs GQ stability and thrombin binding affinity, and the modified TBA can act as a combinatorial logic circuit having INHIBIT logic functions. These attributes make BODIPY-CN a highly useful end-label for creating nanomolecular devices derived from G-rich oligonucleotides.

## Introduction

The development of fluorescent probes that can distinguish different DNA topologies through changes in optical readout has attracted considerable interest due to applications in molecular recognition, biosensing, diagnostics and DNA-based computers^1–4^. One change in topology that is of particular interest involves duplex-quadruplex exchange that is regarded as a nanomolecular device^5^. Compared to the double helix, G-quadruplexes (GQs) are compact, highly polymorphic, may be involved in telomerase inhibition^6^, and are produced by a number of guanine (G)-rich aptamers that bind small molecules^7^, proteins^8^ and metal ion targets^9^ with high affinity and specificity. The fluorescent-based strategies to visualize duplex-GQ exchange include extrinsic “label-free” dyes^10^, covalently attached end-labels^11,12^ and internal-labels that include fluorescent base analogues (FBAs)^13–15^, which are structural mimics of the canonical nucleic acid bases^16^. Our research has focused on the utility of FBAs, particularly 8-aryl-2′-deoxyguanosine (8-aryl-dG) bases, for the development of aptasensors for proteins^14,15^, food toxins^7^ and metal ions^17^. As proof-of-concept for the utility of 8-aryl-dG bases, we commonly employ the 15-mer thrombin binding aptamer (TBA, 5′-GGTTGGTGTGGTTGG)^18^ that folds into a unimolecular antiparallel GQ upon binding thrombin and certain metal ions^19^. Within TBA, 8-aryl-dG bases, such as 8-thienyl-dG^20^, exhibit quenched emission in the duplex that lights-up upon GQ formation due to efficient energy-transfer^21,22^ (up to ~11-fold increase in emission intensity compared to duplex emission^20^). Although FBAs have certain advantages over free dyes and end-labels^16^, a major hurdle for their utility is that they are significantly less bright (εΦ_*fl*_) than commonly used external fluorophores^16^ and have excitation maxima in the UV-region.

In search for brighter visible alternatives capable of discerning duplex from GQs, we were encouraged by the report that the commercial 5′-4,4-difluoro-4-bora-3a, 4a-diaza-s-indacene (BODIPY^®^FL) can distinguish the duplex topology from the single-strand through a photoinduced electron transfer (PET) quenching mechanism of the fluorophore by a nearby G^23^. The best signal-off response (12-fold) occurred with the BODIPY®FL attached to a 5′-C with hybridization to a complementary strand containing a 3′-GGG-overhang that is not base-paired within the duplex. This suggested that a 5′-BODIPY may serve as a useful end-label for monitoring DNA duplex-GQ exchange in the absence of a second label, such as a commercial quencher. These so called quencher-free molecular beacon systems^24^ are particularly advantageous for ease of oligonucleotide synthesis and purification. Furthermore, BODIPYs possess very high quantum yields, are often more photostable than fluorescein (FAM) and rhodamine analogs^25^ and can readily be functionalized at desired positions to improve photophysical properties^26^ and perhaps aptamer performance. This prompted our laboratory to synthesize BODIPY probes to test performance at the 5′-end of TBA in order to make direct comparison to internal FBAs and 5′-FAM^27^. Our results demonstrate the utility of 5′-BODIPY containing cyanophenyl substituents for monitoring duplex-GQ exchange in a quencher-free format. Unlike 5′-FAM^27^, the BODIPY label exhibits minimal impact on GQ stability compared to native TBA^28^ and demonstrates equivalent thrombin binding affinity to that determined for a less-bulky, minimally perturbing, internal FBA^27^. Furthermore, the BODIPY end-label is considerably brighter than internal FBAs used previously by our laboratory^15,27^ and undergoes excitation in the visible region with a reasonably large Stokes shift for aptasensor applications.

## Results and Discussion

### BODIPY-CN Synthesis, Structure and Photophysical Properties

**Figure 1:**
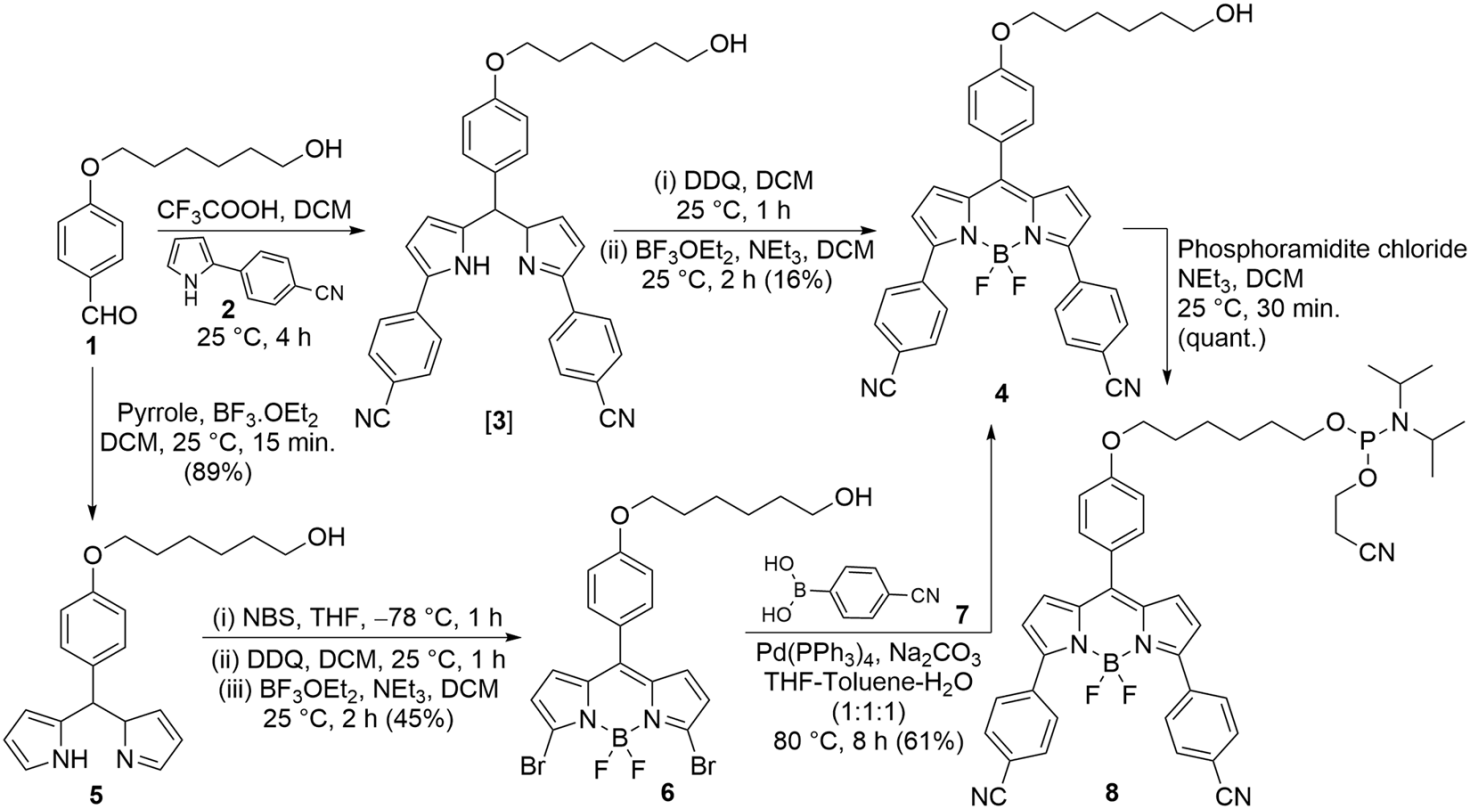
Synthesis of BODIPY-CN alcohol **4** and its phosphoramidite **8**.

BODIPY end-labels used previously for oligonucleotide detection contain alkyl substituents attached to the BODIPY core^23,29^. These analogs possess small Stokes shifts (7–20 nm), which can cause self-quenching^30^. To increase the Stokes shift of the BODIPY end-label, cyanophenyl substituents were attached to the BODIPY core (Fig. 1). The rationale behind this modification was to provide a biphenyl-like structure with differences between the equilibrated geometries and dipole moments in the twisted ground and planar excited states^31^. Furthermore, the electron-withdrawing CN substituents would be expected to enhance photostability^32^ and intramolecular charge transfer (ICT) in the excited state for a larger Stokes shift^33^.

The synthesis of BODIPY-CN alcohol **4** (Fig. 1) was achieved by two different pathways in comparable yields. In the first pathway, 2-(4′-cyanophenyl)pyrrole (**2**)^34^ was utilized in acid-mediated cyclization with aldehyde **1**^35^ followed by oxidation using 2,3-dichloro-5,6-dicyano-1,4-benzoquinone (DDQ) and difluoroborylation using boron trifluoride diethyl ether (BF_3_.OEt_2_) to obtain compound **4** in 16% overall yield. In the second pathway, acid-catalyzed condensation of aldehyde **1** with pyrrole and then bromination at position-2 of both pyrrole rings using N-bromosuccinimide (NBS) provided a dibromo compound that was immediately oxidized using DDQ and treated with BF_3_.OEt_2_ to furnish the BODIPY-dibromide **6**. Finally, Suzuki coupling of **6** with 4-cyanophenylboronic acid **7** provided the BODIPY-CN alcohol **4** in 24% overall yield.

**Figure 2:**
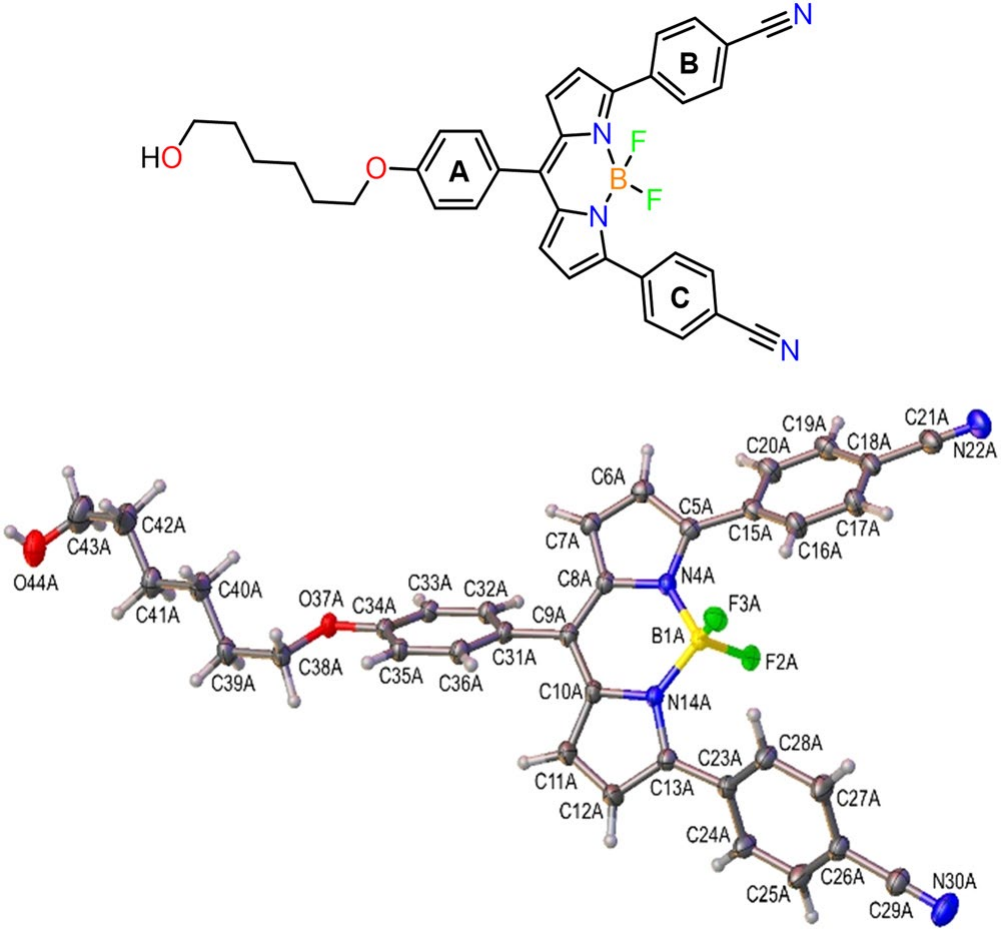
Perspective drawing of the BODIPY-CN alcohol **4** in the crystal with the three phenyl rings attached to the BODIPY core labeled A–C in the schematic.

Crystals of the BODIPY-CN alcohol **4** suitable for X-ray analysis were grown upon slow evaporation of the CHCl_3_ solution at room temperature. The crystal structure demonstrated a high degree of twist between the BODIPY core and the three attached phenyl rings (labelled A–C in schematic, Fig. 2). The A-ring attached to the alcohol linker has a dihedral (twist) angle of 50.6° relative to the BODIPY core, while the corresponding angles for the B- and C-rings are 33.3° and 42.9° in the crystal structure. The fluorine atom F3A (Fig. 2) makes close contact with C16A of the B-ring (3.048 Å) and C28A of the C-ring (3.047 Å) suggesting the formation of two weak intramolecular H-bonds.

**Figure 3:**
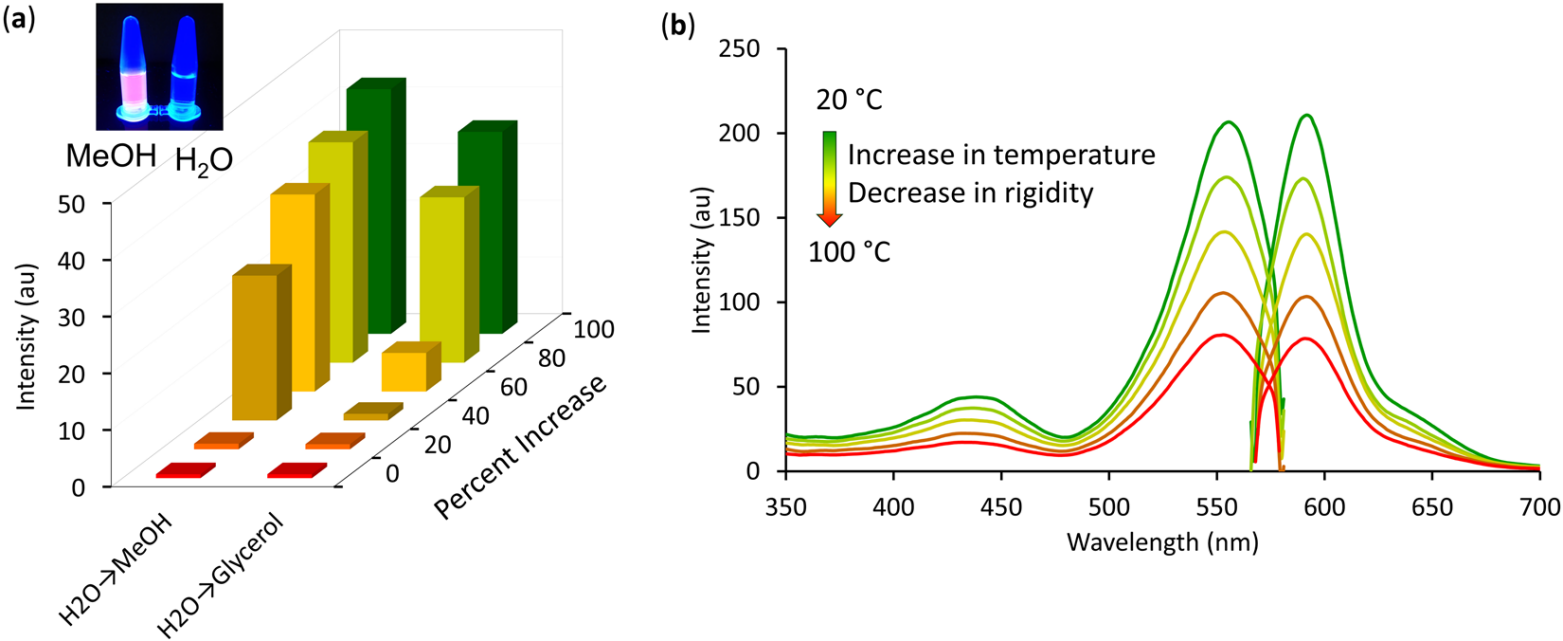
Photophysical properties of the BODIPY-CN alcohol **4**: (**a**) fluorescence response (λ_ex_ = 550 nm, λ_em_ = 586 nm) of 4 (1 μM) in water with increasing percentages of methanol and glycerol; inset, fluorescence images of 4 in methanol versus water, (**b**) fluorescence response of 4 (1 μM) as a function of temperature in methanol:glycerol (1:4).

In methanol the BODIPY-CN alcohol **4** exhibited λ_max_ at 550 nm (ε = 80,745 M^−1^. cm^−1^) with emission at 586 nm (Stokes shift, Δ*v* = 36 nm) with a relative quantum yield (Φ_*fl*_) of 0.58 (using rhodamine 101 (Φ_*fl*_ = 1) as a standard) for a brightness (εΦ_*fl*_) of 46,832 M^−1^. cm^−1^. The BODIPY-CN dye exhibited weak solvatochromic properties, displaying little changes in λ_ex_/λ_em_ in solvents of different polarity (Supporting Information, Fig. S1), but displayed quenched emission in water (Φ_*fl*_ = 0.21, brightness ~16,956 M^−1^. cm^−1^) compared to its emission in methanol that was ascribed to self-aggregation (Fig. 3a)^36^. The probe was also found to exhibit enhanced emission with increased solvent viscosity (Fig. 3a) that was diminished with increased temperature (Fig. 3b).

### Duplex-Quadruplex Exchange

To attach the BODIPY-CN chromophore to the 5′-end of TBA, the alcohol **4** was converted into the phosphoramidite **8** (Fig. 1) for solid-phase DNA synthesis using UltraMild conditions; a requirement for oligonucleotide synthesis involving the BODIPY chromophore^29^. In the previous quencher-free assay using 5′- BODIPY®FL end-label the best signal-off response occurred with the BODIPY attached to a 5′-C with hybridization to a complementary 3′-G to generate duplex structures^23^. The BODIPY quenching was especially efficient (12-fold) when the complementary strand contained a 3′-GGG-overhang that is not base-paired within the duplex. For TBA the 5′-base is G and so it was reasoned that the 5′-BODIPY-CN-TBA would show quenched emission in the strand or GQ structure that may exhibit a signal-on response upon duplex formation with the 5′-G base-paired to C. To test this hypothesis the fluorescence response of the BODIPY-CN-TBA sample was recorded in aqueous buffer in the absence and presence of a truncated 10-mer complementary strand 5′-CACACCAACC (CS-10) utilized previously by our laboratory to monitor duplex-GQ exchange by mTBA containing internal FBAs^13^.

**Figure 4:**
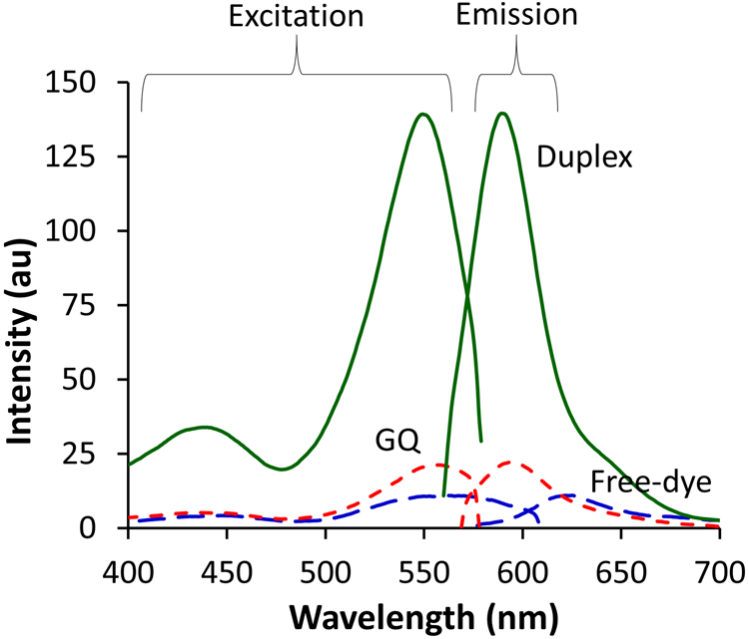
Overlay of excitation and emission spectra of BODIPY-CN alcohol **4** (Free-dye (1.75 μM), dashed blue trace) versus BODIPY-CN-TBA (1.75 μM) in the absence (GQ, dashed red trace) and presence of CS-10 (1.5 equiv., Duplex, solid green trace).

**Figure 5:**
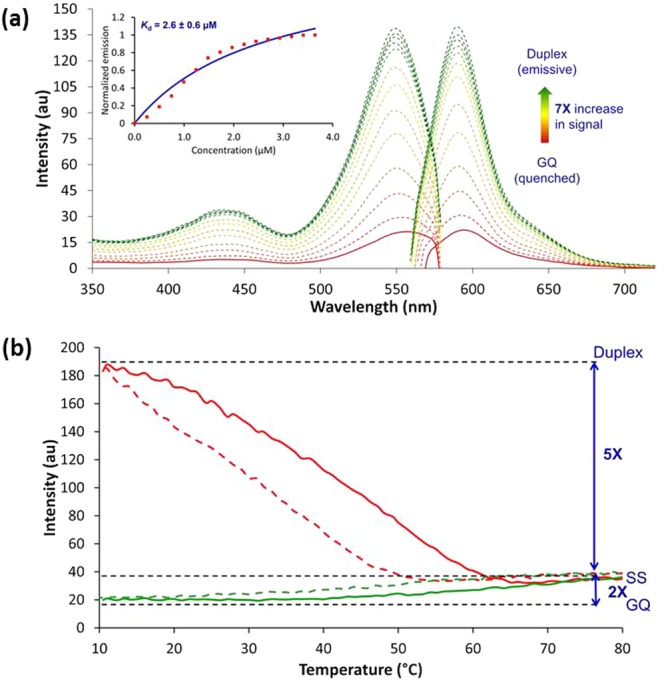
(**a**) Fluorescence titration of BODIPY-CN-TBA (1.75 μM) with CS-10 at 21 °C; initial trace of BODIPY-CN-TBA depicted by the solid red line, while dashed traces depict duplex formation upon successive addition of CS-10; insert plot of the fluorescence intensity versus [CS-10]. (**b**) Fluorescence thermal melting analysis of BODIPY-CN-TBA (1.75 μM) in the absence (green traces) and presence (red traces) of 1.5 equiv. CS-10; heating ramps are solid lines, cooling ramps are dashed.

As shown in Fig. 4, the free-dye alcohol **4** displays a broad excitation spectrum with emission peaking at 622 nm (dashed blue trace, Fig. 4). The BODIPY-CN-TBA sample (dashed red trace, Fig. 4) exhibits emission at 595 nm (λ_ex_ = 557 nm) for a blue-shift of 27 nm compared to the free-dye that was also accompanied with a 2-fold increase in intensity. These changes were consistent with self-aggregation by the free BODIPY **4** that was disrupted by attaching it to the 5′−end of TBA. In the presence of 1.5 equiv. of CS-10 the emission intensity increased significantly (7-fold compared to the unpaired BODIPY-CN-TBA, 14-fold compared to the free-dye **4**) with emission at 590 nm (λ_ex_ = 550 nm, solid green trace, Fig. 4). These results suggested that addition of CS-10 promoted duplex formation that inhibited PET quenching of the BODIPY chromophore by the 5′-G. To test this hypothesis in more detail, CS-10 was titrated into an aqueous solution containing BODIPY-CN-TBA and a dose-dependent increase in emission intensity at 590 nm was observed (Fig. 5a). A plot of the fluorescence intensity versus [CS-10] indicated a 1:1 CS-10:BODIPY-CN-TBA interaction and afforded a dissociation constant (*K*_d_) of 2.6 ± 0.6 μM (insert, Fig. 5a). Fluorescence thermal melting analysis also demonstrated the emission sensitivity of the CS-10:BODIPY-CN-TBA sample to heating and cooling, which was consistent with thermal denaturation of the emissive duplex into the single-strand with quenched emission (Fig. 5b).

Overall, a 10-fold increase in emission intensity was observed for the duplex versus the GQ at 10 °C (Fig. 5b). These results were similar to our previous studies involving the photophysical properties of the FBA 8-(4″-cyanophenyl)-dG (^CNPh^dG) containing the cyanophenyl substituent directly attached to the C8-site of dG^13^. Within TBA as replacement for G_8_^CNPh^, dG displayed enhanced emission intensity within the duplex that was strongly quenched (10-fold) upon GQ formation. Given that DNA-to-probe energy transfer efficiencies are much higher in GQ structures than the same oligonucleotides folded in B-form duplexes^22^, quenching of the ^CNPh^dG emission in the GQ was ascribed to PET from the electron-rich G-tetrad into the electron-deficient nucleobase^13^. Thus, the photophysical behavior of mTBAs containing 5′-BODIPY-CN or ^CNPh^dG is contrasted by that of most internal FBAs that exhibit strongly quenched fluorescence in the duplex topology^16^. In fact, one of the brightest FBA in duplex DNA is a pentacyclic adenine (pA) with a brightness of only 1400 M^−1^cm^−1^ ^16^. That the BODIPY-CN-TBA duplex exhibits a 14-fold increase in emission intensity compared to the free-dye **4** that possesses a brightness of ~16,956 M^−1^. cm^−1^ highlights the potential sensitivity of BODIPY-CN for distinguishing duplex from GQ structures.

### GQ Formation and Thrombin Binding

**Figure 6:**
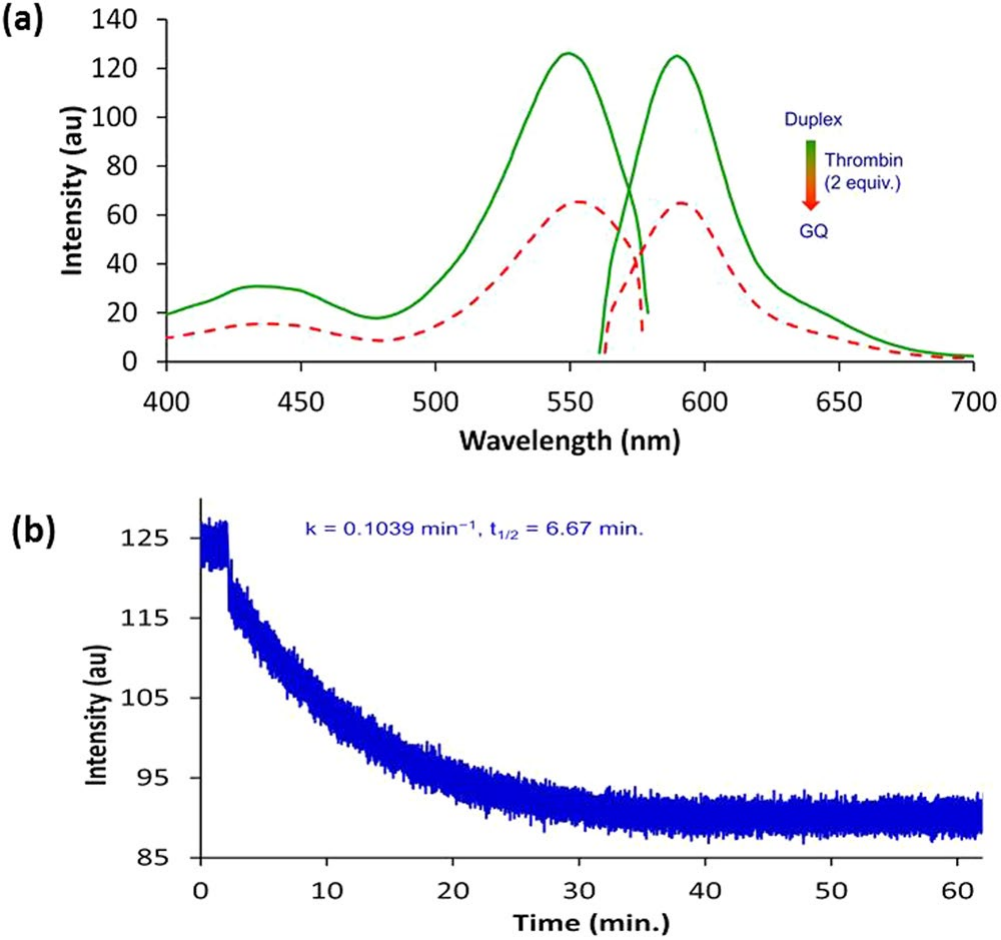
(**a**) Fluorescence overlay spectra of BODIPY-CN-TBA:CS-10 (1.75 μM) in the absence (solid green trace) and presence of 2 equiv. thrombin (dashed red trace). (**b**) Fluorescence emission (λ_ex_ = 550 nm; λ_em_ = 590 nm) intensity trace as a function of time for BODIPY-CN-TBA:CS-10 (1.75 μM) in the presence of 2 equiv. thrombin.

Formation of the antiparallel GQ by the BODIPY-CN-TBA sample was supported by CD spectral analysis and UV thermal melting at 295 nm. Unmodified TBA displays a characteristic antiparallel GQ CD spectrum with positive peaks at 290 and 240 nm with a negative peak at 260 nm with a thermal melting temperature (*T*_m_) of ~53.5 °C^27^. The BODIPY-CN-TBA sample afforded a *T*_m_ of 51.0 °C and provided the characteristic antiparallel GQ CD spectrum (Fig. S2). In the presence of 1.5 equiv. CS-10, a B-form duplex CD spectrum with roughly equal positive (275 nm) and negative (244 nm) bands with a crossover at ~260 nm was observed (Fig. S2)^37^. The truncated duplex had a *T*_m_ of 38.0 °C, as monitored by UV at 260 nm. The diminished *T*_m_ of the truncated duplex compared to the GQ (Δ*T*_m_ = 51–38 = 13 °C) suggested the ability to utilize the pre-formed duplex to monitor thrombin binding, as liberation of the GQ from CS-10 promoted by thrombin would quench BODIPY-CN fluorescence to signal target binding^20^. This ability was initially evaluated by adding 2 equiv. of thrombin to the duplex sample (Fig. 6). Addition of thrombin caused a 2-fold reduction in the emission of the duplex (Fig. 6a), suggesting GQ formation with liberation of CS-10. A fluorescence emission trace as a function of time provided an apparent first-order thrombin binding rate constant *k*_obs_ of 0.1049 min^−1^ for a half-life (*t*_1/2_) of 6.67 min (Fig. 6b).

A fluorescence titration carried out with thrombin and the truncated duplex provided a *K*_d_ value of 1.2 μM for protein binding to BODIPY-CN-TBA (Fig. S3). For comparison, thrombin binding affinity of the BODIPY-CN-TBA strand in the absence of CS-10 was also measured using fluorescence polarization (FP, Fig. S3) and the *K*_d_ (1.3 μM) was within experimental error of the value determined from duplex-GQ exchange. The impact of the BODIPY-CN chromophore on GQ stability and thrombin binding affinity is summarized in Table 1. For direct comparison to the 5′-BODIPY-CN probe, previous data determined for native TBA^28^, 5′-FAM-labeled TBA^27^ and mTBA containing C8-(4-cyanophenyl-vinyl)-dG (^CN^dG) at G_6_ within the G-tetrad^27^ are included. The binding data for native TBA was determined using isothermal titration calorimetry (ITC) and generated an apparent binding constant *K*_b_ = 3 × 10^6^ M^−1^, for a dissociation constant *K*_d_ = 0.33 μM^28^. The modifications decrease the *T*_m_ of the GQ and increase *K*_d_. However, it is clear that the 5′-BODIPY-CN probe developed in this work is far less perturbing than the commercially available 5′-FAM label that strongly decreases GQ stability (Δ*T*_m_ = −9.5 °C) and thrombin binding affinity (*K*_d_ = 4.9 μM)^27^. These changes cannot be ascribed to the nature of the linker, as both dyes are attached to the 5′-end of TBA using a six-carbon tether. Instead, the data highlights the diverse impact that a 5′-dye can have on aptamer structure and function. Furthermore, the 5′-FAM lacks fluorescence sensitivity to duplex-GQ exchange and the binding affinity was determined using FP^27^. In fact, the *T*_m_ and *K*_d_ values recorded for 5′-BODIPY-CN-TBA resemble the corresponding values obtained for mTBA with the internal FBA^CN^, dG, that typically has lower interference during interaction between the aptamer and target^27^. However, similar to the 8-styryl-dG (^Sty^dG) nucleobase^22,27^, the brightness of the internal ^CN^dG probe is only ~(0.2 (Φ_*fl*_) × 25,000 (ε_max_) = 5200 M^−1^ cm^−1^) in duplex DNA. Thus, given the superior brightness of the BODIPY-CN probe, coupled with its minimal impact on GQ-folding and thrombin binding by TBA, it possesses greater potential than other FBAs including ^CN^dG for utility within fluorescent aptasensors involving G-rich oligonucleotides.

**Table 1:** Fluorescence, UV-thermal melting parameters and dissociation constants for thrombin binding by native TBA and mTBA samples.

label	λ_ex_ (nm)	λ_em_ (nm)	*T*_m_ (Δ*T*_m_) (°C)	*K*_d_ (μM)
Native	—	—	53.5 (0)	0.33
5′-BODIPY-CN	557	595	51.0 (−2.5)	1.2 ± 0.1 (1.3)
5′-FAM	495	520	44.0 (−9.5)	4.9 ± 0.1
G_6_-^CN^dG	378	501	51.5 (−2.0)	1.9 ± 0.1

### Logic Gate Construction

**Figure 7:**
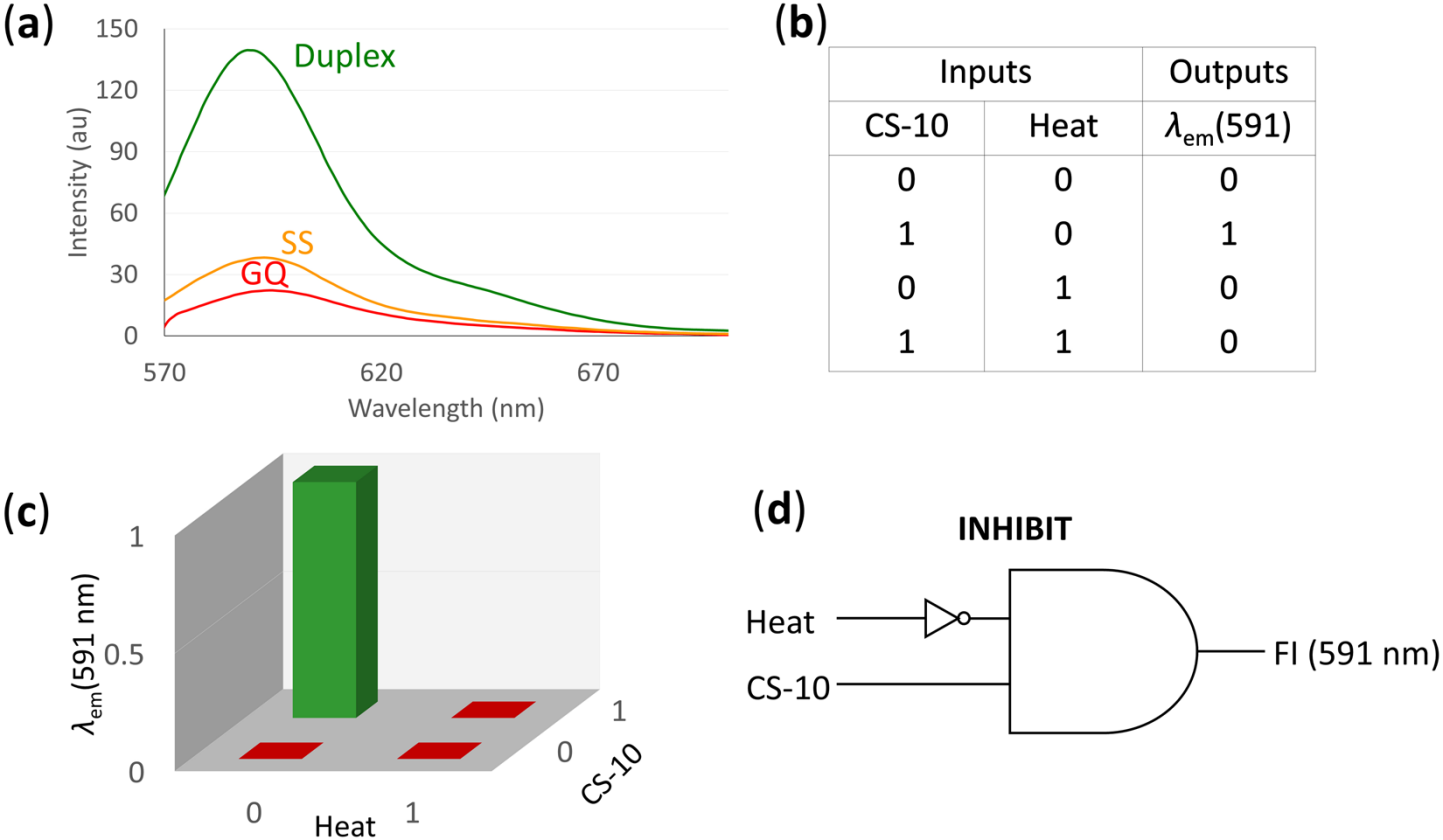
(**a**) Fluorescence emission spectra of BODIPY-CN-TBA at different input conditions, namely GQ, single strand and duplex DNA; (**b**) the truth table for sequential logic circuit, where ‘0’ = ‘Off’ and ‘1’ = ‘On’ signals; (**c**) fluorescence emission spectra of BODIPY-CN-TBA at different inputs according to the truth table (**b**). Fluorescence intensities higher and lower than the threshold value (50) at 590 nm are assigned as ‘1’ and ‘0’ respectively; and (**d**) INHIBIT logic gate has been constructed based on results obtained in (**a**,**b**) and (**c**).

With the recent trend of converting chemically encoded information into fluorescence signals^4^, a combinatorial logic device was envisioned for the BODIPY-CN-TBA system. It was constructed using two inputs, namely heat and CS-10, with the fluorescence signal obtained from BODIPY-CN-TBA as an output. The threshold intensity of 50 au was chosen at a fluorescence output of 590 nm (Fig. 7a). Fluorescence intensity higher than the threshold value is assigned as “1”, and lower than that is assigned as “0” signifying the “signal-on” and “signal-off” states, respectively. The truth table (Fig. 7b) was drawn for various combinations and sequential addition of these two chemical inputs, where the fluorescence intensity output at 590 nm is read as ON (1) or OFF (0) (Fig. 7c). Based on the output values in truth table, a INHIBIT logic gate was constructed (Fig. 7d). The combination of these properties coupled with selectivity by different inputs allows its implementation to design a simple molecular switch.

## Conclusions

In summary, we have designed and synthesized a novel BODIPY derivative as a 5′-end-label for solid-phase oligonucleotide synthesis. The free-dye BODIPY-CN alcohol **4** possesses a Stokes shift of ~40 nm with λ_ex_ = 550 nm and λ_em_ = 586 nm with a brightness (εΦ_*fl*_) of 46,832 M^−1^ cm^−1^ in methanol that is reduced to ~16,956 M^−1^ cm^−1^ in aqueous buffer through self-aggregation. When attached to the 5′-end of the 15-mer TBA that contains the G nucleobase, the probe exhibits ~2-fold increase in fluorescence intensity compared to the free-dye in aqueous buffer. However, when the 5′-G is base-paired to a complementary C, the emission of the BODIPY-CN probe increases 7-fold, 14-fold compared to the free-dye. This emissive switching mechanism is ascribed to PET quenching by the 5′-G in TBA that is inhibited in the duplex structure. DNA-to-probe energy transfer efficiencies are known to be much higher in GQ structures than the same oligonucleotides folded in B-form duplexes^22^ and this enables the BODIPY-CN end-label to serve as a quencher-free visible fluorescent probe for monitoring duplex−GQ exchange for applications in DNA-based diagnostics.

## Methods

### Materials and Methods

The cartridge purified native TBA, CS-10 and FAM-TBA were purchased from Sigma-Aldrich Ltd. (Oakville, ON). Reagents for UltraMild oligonucleotide synthesis of TBA containing the BODIPY-CN 5′-end-label were purchased from Glen Research (Sterling, VA). Oligonucleotide synthesis was carried out at 1 μmol using a MerMade 12 DNA synthesizer and deprotection of the BODIPY-CN-TBA sample was carried out for 4 hours in 0.05 M potassium carbonate in methanol. Bovine thrombin was purchased from BioPharm Laboratories LLC (Bluffdale, Utah). Commercially available pyrrole, trifluoroacetic acid, BF_3_.OEt_2_, NBS, DDQ, 4-cyanophenylboronic acid and 2-cyanoethyl *N*,*N*-diisopropylchlorophosphoramidite were used as received. NMR spectra were recorded on 300 or 400 MHz spectrometers in CDCl_3_ referenced to TMS (0 ppm) or CHCl_3_ (7.25 ppm). All UV-vis spectra were recorded with baseline correction and stirring using 10 mm light path quartz glass cells. Any water used for buffers or spectroscopic solutions was obtained from a filtration system (18.2 MΩ). High-resolution mass spectra (HRMS) for compounds **4–6** (Fig. 1) were recorded on a Q-Tof instrument, operating in electrospray ionization (ESI) at 5–10 μL/min detecting positive ions. Phosphoramidites are air sensitive compounds^22^, and the BODIPY-CN phosphoramidite **8** was characterized by NMR (^1^H and ^31^P), and then immediately carried into oligonucleotide synthesis. Full synthetic details are available in Supporting Information.

### X-Ray crystallography

For the crystal structure analysis, the BODIPY-CN dye **4** was crystallized from chloroform. A red plate with dimensions 0.50 × 0.25 × 0.04 mm was mounted on a MiTeGen MicroMount holder and cooled down to 150 K. All X-ray diffraction measurements were conducted at this temperature. The crystal was studied on a SuperNova single-crystal diffractometer equipped with a microfocus Cu*K*_α_ (λ = 1.54184 Å) radiation source, Atlas CCD detector and CryoJet low-temperature device. Diffraction intensity data were collected using ω-scan to the maximum 2θ angle of 151.7° (resolution of 0.795 Å), with the redundancy factor of >11. The unit cell parameters were refined using the entire data set. The data were processed using CrysAlisPro software. Absorption corrections were applied using the multiscan method. The structure was solved (direct methods) and refined (full-matrix least-squares on *F*^2^) using SHELXS^38^ and SHELXL-2013^39^. Non-hydrogen atoms were refined anisotropically, except for the oxygen atom of solvent water, which was refined isotropically without hydrogen atoms. All hydrogen atoms were refined isotropically with free coordinates, except for the hydrogen atoms of the solvent chloroform and the disordered -CH_2_OH group of the BODIPY-CN dye. Geometric calculations were carried out using the WinGX^40^ and Olex^41^ software packages. The crystal structure data have been deposited with the Cambridge Crystallographic Data Centre (deposition no. 1828909) and a copy of these data are available free of charge upon request from the CCDC web-site: http://www.ccdc.cam.ac.uk/data_request/cif or by e-mail: deposit@ccdc.cam.ac.uk.

### Oligonucleotide purification and characterization

The crude BODIPY-CN-TBA sample was suspended in Milli-Q water (18.2 MΩ) and purified using an Agilent HPLC instrument equipped with an autosampler, a diode array detector (monitored at 258 and 550 nm), fluorescence detector (monitored at λ_ex_ = 550 nm and λ_em_ = 591 nm), and autocollector. Purification was carried out at 50 °C using a 5 μm reversed-phase (RP) semipreparative C18 column (100 × 10 mm) with a flow rate of 3.5 mL/min, and various gradients of buffer B in buffer A (buffer A = 19:1 aqueous 50 mM TEAA, pH 7.2/acetonitrile; buffer B = 3:7 aqueous 50 mM TEAA, pH 7.2/acetonitrile). Collected DNA samples were lyophilized to dryness and redissolved in 18.2 MΩ water for quantification by UV-vis measurement using ε260. Extinction coefficients were obtained from the following website: http://www.idtdna.com/analyzer/applications/oligoanalyzer. Mass of the 5′−BODIPY-CN-TBA was acquired on a Bruker AmaZon quadrupole ion trap SL spectrometer in the negative ESI mode. The oligonucleotide sample was prepared in 90% Milli-Q filtered water/10% methanol containing 0.1 mM ammonium acetate. Full scan MS spectra were obtained by direct infusion at a rate of 5–10 μL/min.

### UV thermal denaturation and CD studies

All melting temperatures (*T*_m_’s) of 5′-BODIPY-CN-TBA samples were measured on a Cary 300-Bio UV-Vis spectrophotometer at a concentration of 3.0 μM in 100 mM M^+^–phosphate buffer (pH 7.0) with 0.1 M M^+^Cl where M^+^ = K^+^ or Na^+^. Duplex samples were prepared with 1.5 equivalents of 10-mer complementary strand (CS-10) purchased from Sigma-Aldrich and used without further purification. The UV absorption was monitored as a function of temperature at either 295 nm for GQ, or 260 nm for duplex, and consisted of forward-reverse scans from 10 to 90 °C at a heating rate of 0.5 °C/min, and was repeated at least three times. The *T*_m_ values were calculated by determining the first derivative of the melting curve through the Varian Thermal software. CD spectra were performed on a Jasco J-815 CD spectrophotometer equipped with a thermally controlled 1 × 4 multicell block. The annealed samples obtained from the *T*_m_ studies were measured at 10 °C in quartz cells (110-QS) with a light path of 1 mm and monitored between 200 and 400 nm at a bandwidth of 1 nm and scanning speed of 100 nm/min.

### Fluorescence, titrations and kinetic measurements

The fluorescence of the annealed 5′-BODIPY-CN-TBA samples were measured on a Cary Eclipse Fluorescence spectrophotometer as both excitation and emission spectra in quartz cells (108.002F-QS) with a path length of 10 mm at 21 °C. Excitation and emission slit widths were kept constant at 5 nm. CS-10 titrations with 5′-BODIPY-CN-TBA (1.75 μM) pre-folded into the GQ in 50 mM potassium phosphate buffer, pH 7.1, 100 mM KCl were carried out with additions of 2 μL CS-10 from a 125 mM stock solution in water until a final concentration of 3-4 equiv. of CS-10 to the GQ was reached. Thrombin titrations were performed on duplex samples at a concentration of 1.75 μM 5′-BODIPY-CN-TBA in 50 mM potassium phosphate buffer pH 7.0 with 0.1 M KCl with 1.5 equivalents of CS-10. Titrations proceeded according to a previously published protocol^20^ with additions of 1 μL of a 50 μM thrombin protein solution in 50 mM sodium phosphate buffer (pH 7.0) with 0.1 M NaCl until a final concentration of two equivalents of protein had been added. For both titrations, scans were taken 10 minutes after addition of the protein or CS-10. Plots of the fraction of aptamer bound versus [thrombin or CS-10] generated binding isotherms that were analyzed with SigmaPlot 13.0 to obtain *K*_d_ values. The FP titrations proceeded according to previously published protocols with minor variations^20,27^. Samples of 5′-BODIPY-CN-TBA (1 μM) were prepared in 50 mM potassium phosphate buffer, pH 7.1, 100 mM KCl in a 1 mL quartz cell (114F-QS). Titrations were run on a PTI QuantaMaster Fluorimeter at 21 °C and measured at λ_ex_ = 551 nm, λ_em_ = 591 nm with slit widths of 5 and 10 nm, respectively. Kinetic measurements for thrombin binding to 5′−BODIPY-CN-TBA annealed to CS-10 (1.5 μM duplex) were carried out as previously described for other modified TBA samples^20^.

## Electronic supplementary material

Supporting Information
