# Supplementary material for: A 5′-BODIPY End-label for Monitoring DNA Duplex-Quadruplex Exchange
Source: Sci Rep. 2018 Nov 15;8:16874. doi: 10.1038/s41598-018-35352-0 (PMC6237993; doi:10.1038/s41598-018-35352-0)
Supplement: Supplementary file 1 — Supporting Information [file 41598_2018_35352_MOESM1_ESM.pdf]

Supporting Information for:

A 5′−BODIPY End-label for Monitoring DNA Duplex−Quadruplex Exchange

Prashant S. Deore, Dmitriy V. Soldatov and Richard A. Manderville*

Table of Contents:

1. **Figure S1**. Solvatochromic properties of BODIPY-CN alcohol **4**. S2

2. **Figure S2.** CD spectral overlay for BODIPY-CN-TBA. S2

3. **Figure S3**. Thrombin titration data. S3

4. Synthesis of **4** and **8**. S4

5. MS data of BODIPY-CN-TBA S11

6. X-Ray crystallographic data for BODIPY-CN alcohol **4**. S12

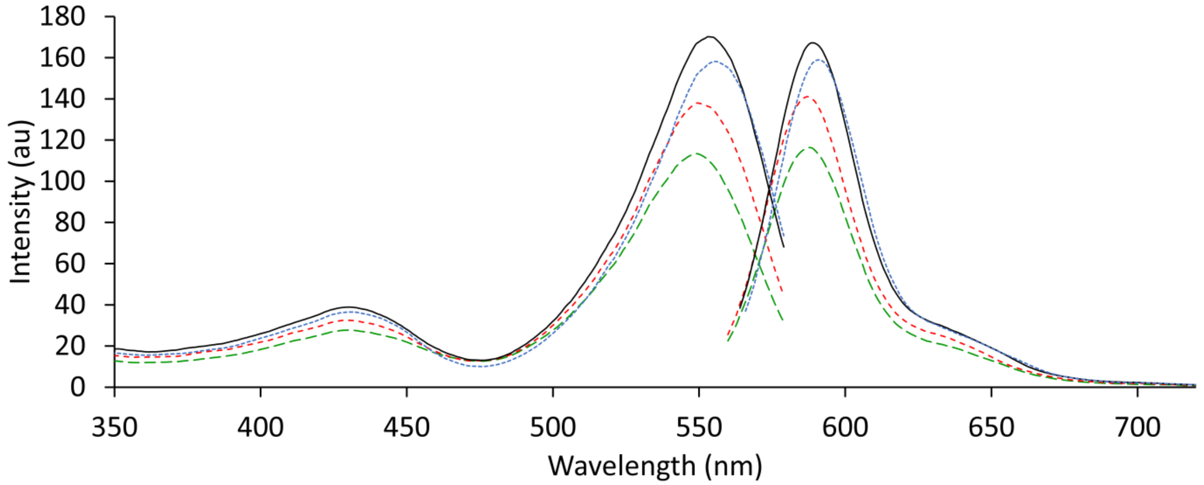

Figure S1. Overlay of excitation and emission spectra of BODIPY-CN alcohol **4** in isopropyl alcohol (solid black trace), $CHCl_3$ (dotted blue trace), methanol (dashed red trace) and CH_3CN (dashed green trace).

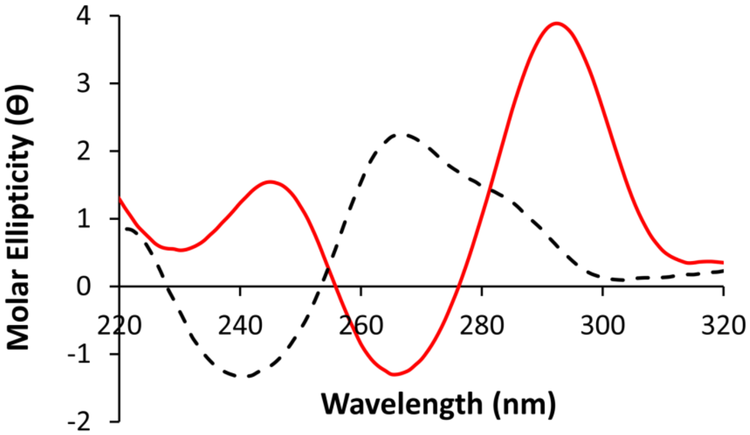

Figure S2. CD spectral overlay of BODIPY-CN-TBA (3 μM) in the absence (solid red trace) and presence of 1.5 equiv. CS-10 (dashed black trace). Solid red trace is characteristic of the antiparallel GQ produced by TBA, while the dashed black trace is characteristic of a B-form duplex.

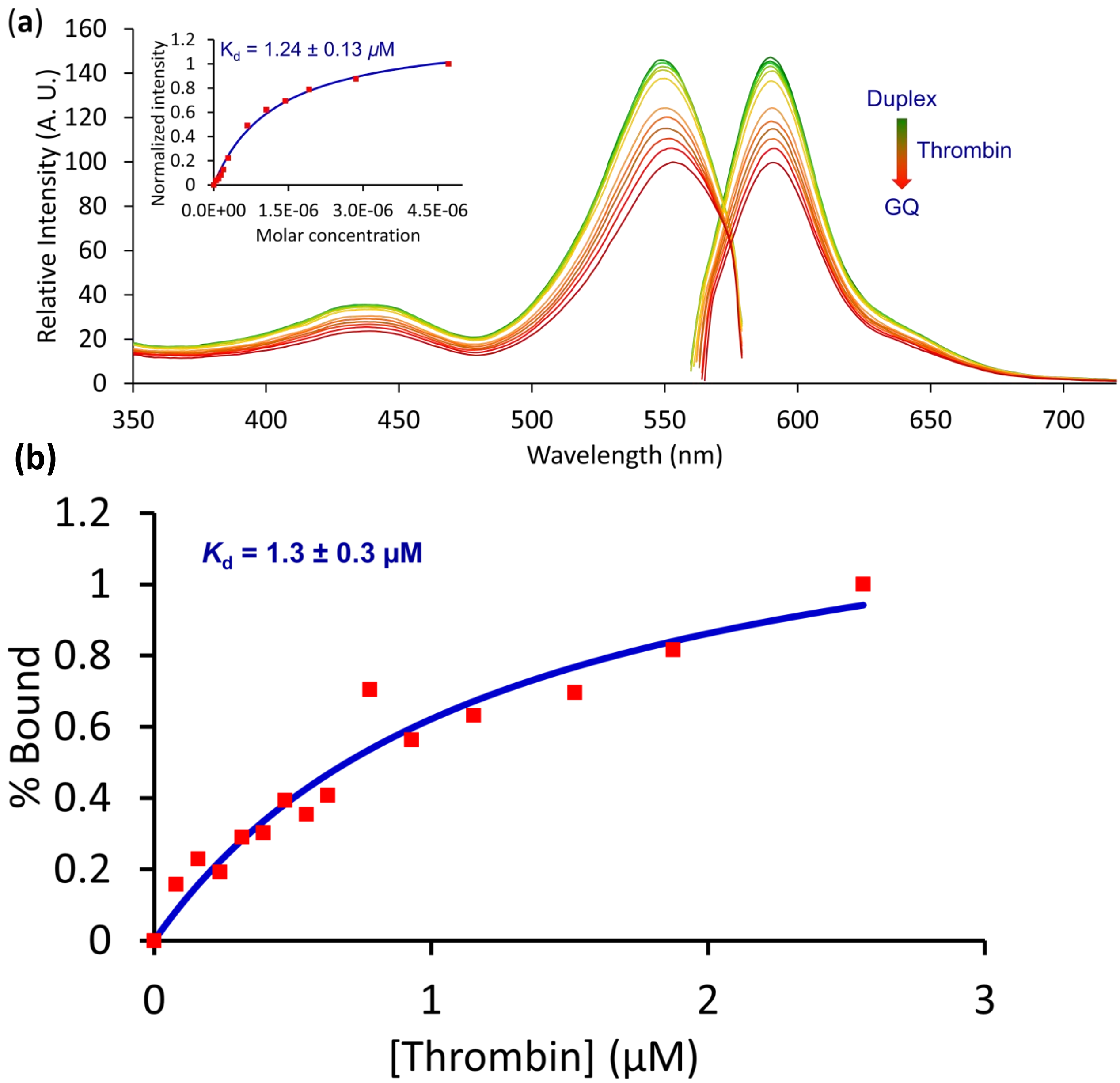

Figure S3.(a) Fluorescence titration of BODIPY-CN-TBA:CS-10 (1.75 μM) with thrombin at 21 °C; initial trace of BODIPY-CN-TBA depicted by the solid green line, while yellow-red lines depict successive addition of thrombin up to 2 equiv. (b) Fluorescence polarization (FP) titration for BODIPY-CN-TBA (1 μM) binding to thrombin at 21 °C.

Synthesis of BODIPY-CN alcohol 4 and its phosphoramidite 8

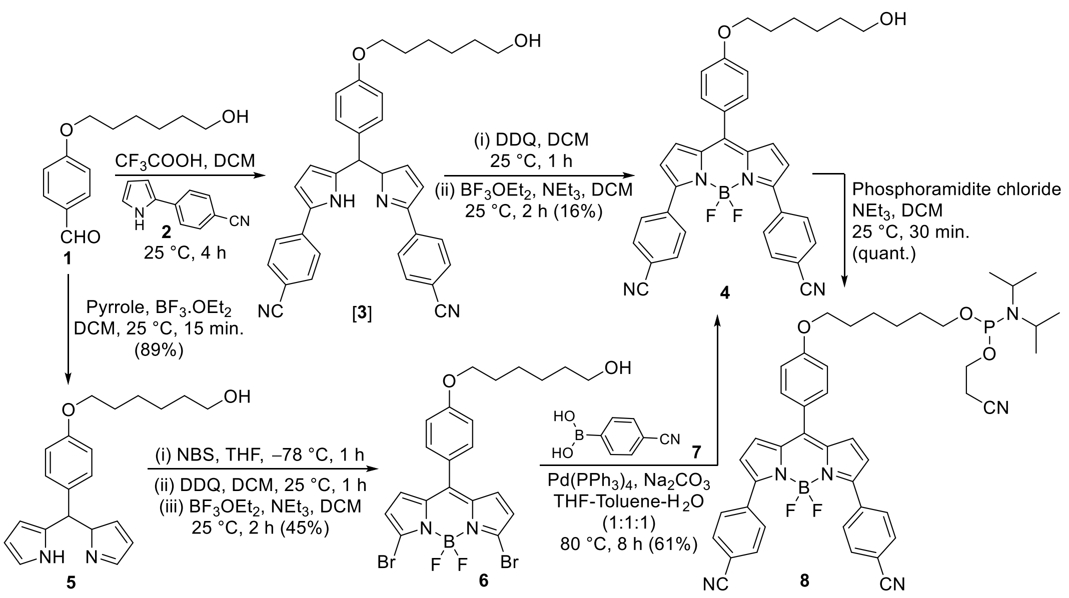

4-(6-Hydroxyalkyl-1-oxy)benzaldehyde (1).

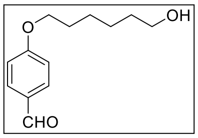

1 was prepared by reported method as an oily compound.[1]

^{1}H NMR ($CDCl_3$, 300 MHz): δ (ppm) 1.36−1.58 (m, 5H), 1.62 (quint., J = 6 Hz, 2H), 1.84 (quint., J = 6 Hz, 2H), 3.67 (t, J = 6 Hz, 2H), 4.05 (t, J = 6 Hz, 2H), 6.99 (d, J = 9 Hz, 2H), 7.83 (d, J = 9 Hz, 2H), 9.88 (s, 1H).

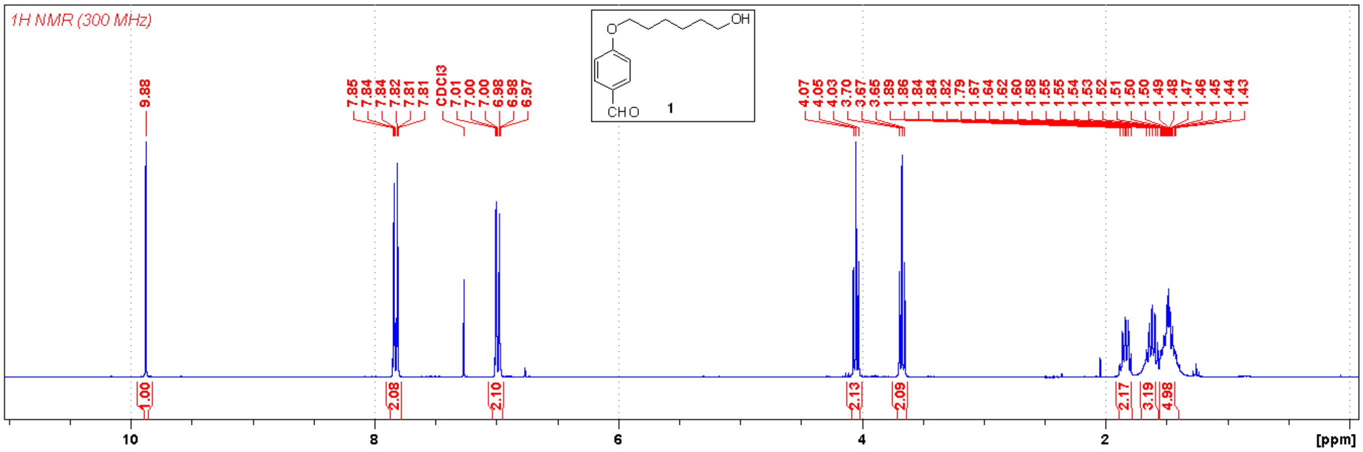

4-(1*H*-Pyrrol-2-yl)benzonitrile (2).

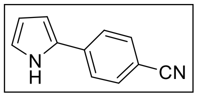

2 was prepared by reported method as a pale yellow solid.[2]

^{1}H NMR ($CDCl_3$, 300 MHz): δ (ppm) 6.35 (m, 1H), 6.68 (m, 1H), 6.96 (m, 1H), 7.55 (d, J = 6 Hz, 2H), 7.63 (d, J = 6 Hz, 2H), 8.71 (br s, 1H).

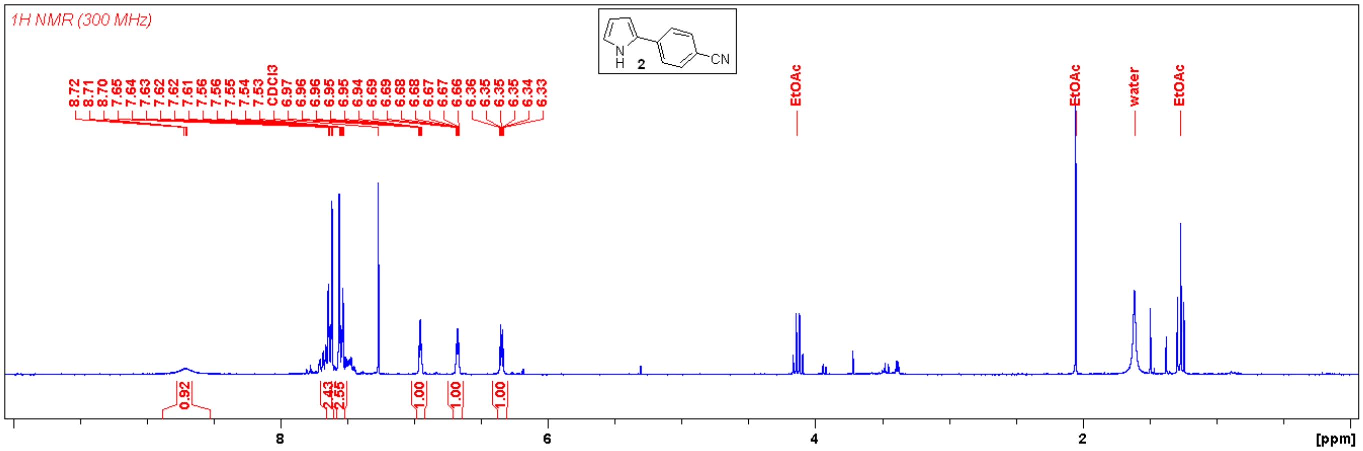

6-(4-(Di(1*H*-pyrrol-2-yl)methyl)phenoxy)hexan-1-ol (5).

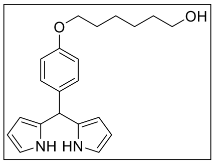

To a round bottomed flask containing aldehyde **1** (400 mg, 1.80 mmol) in dry dichloromethane (20 mL) was added pyrrole (0.623 mL, 9.00 mmol) and catalytic amounts of $BF_3{\cdot}Et_2O$ (3 drops). The mixture was stirred at room temperature for 15 min. under argon atmosphere. After completion of the reaction, the solution was concentrated to dryness under reduced pressure and the crude product was purified by column chromatography (silica gel; EtOAc/hexane) as an off white colored solid (542 mg, 89%).

^{1}H NMR ($CDCl_3$, 300 MHz): δ (ppm) 1.39−1.58 (m, 5H), 1.61 (quint., J = 6 Hz, 2H), 1.81 (quint., J = 6 Hz, 2H), 3.64 (t, J = 6 Hz, 2H), 3.96 (t, J = 6 Hz, 2H), 5.41 (s, 1H), 5.86−5.96 (m, 2H), 6.17 (dd, J = 6 and 1 Hz, 2H), 6.68 (dt, J = 3 and 1 Hz, 2H), 6.86 (d, J = 9 Hz, 2H), 7.13 (d, J = 9 Hz, 2H), 8.02 (s, 2H).

^{13}C NMR ($CDCl_3$, 75 MHz): δ (ppm) 25.4, 25.8, 29.2, 32.5, 43.1, 62.8, 67.8, 106.9, 108.2, 114.5, 117.1, 129.3, 132.9, 134.0, 157.9.

HRMS Calc. for $C_{21}H_{27}N_2O_2^+$ $[M + H]^+$ 339.2073; Found 339.2092.

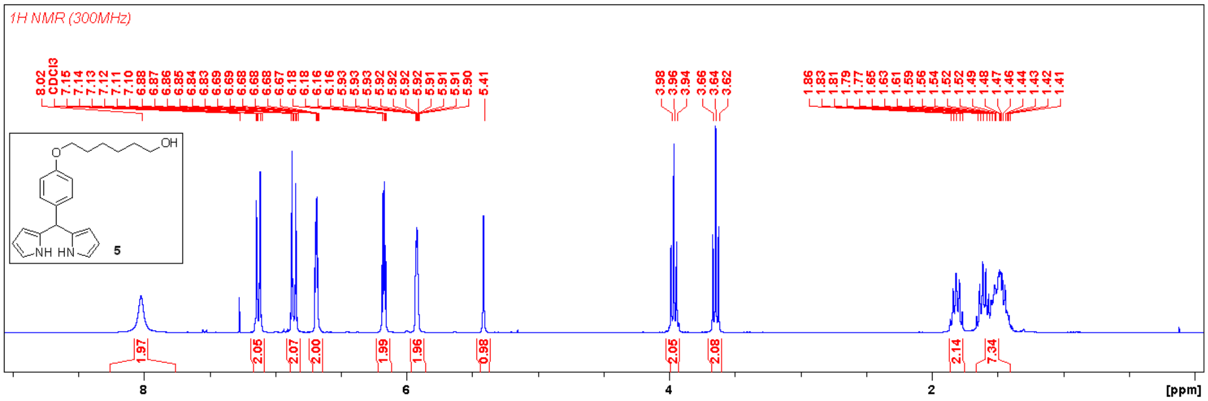

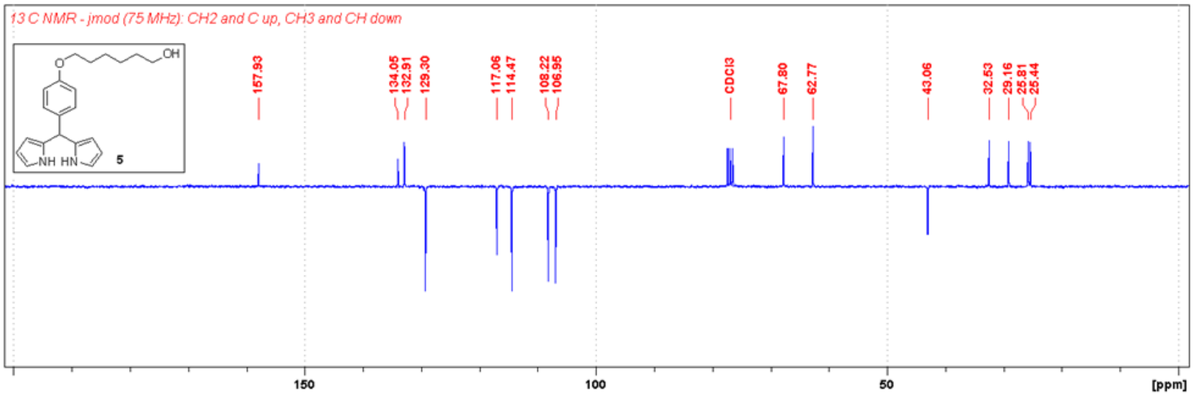

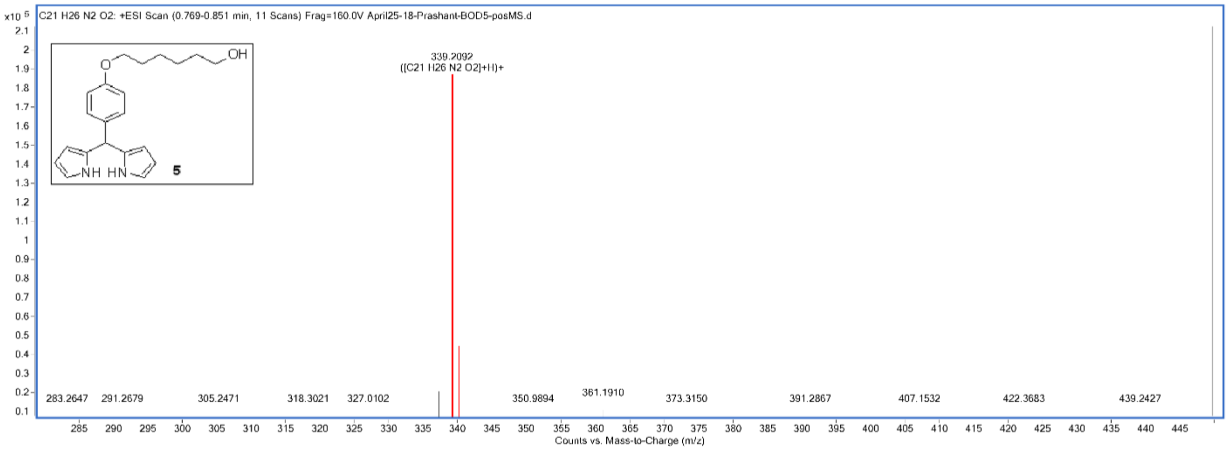

BODIPY-dibromide (6).

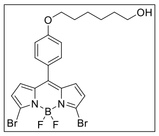

To a stirred solution of **5** (500 mg, 1.48 mmol) in dry THF (25 mL) at −78 °C, NBS (578 mg, 3.25 mmol) was added in three portions (20 min/addition) and the reaction mixture was allowed to stir for an additional

1 h. After completion, the reaction mixture was filtered through a bed of celite and silica gel, and the filtrate was concentrated under reduced pressure to obtain a crude product that was immediately treated with DDQ (336 mg, 1.48 mmol) in dry DCM (mixture allowed to stir for 1 hour) followed by $BF_3 \cdot Et_2O$ (3 mL) and then NEt_3 (0.75 mL) to furnish **6** as a red solid (360 mg, 45%).

^{1}H NMR ($CDCl_3$, 300 MHz): δ (ppm) 1.32−1.58 (m, 5H), 1.64 (quint., J = 6 Hz, 2H), 1.87 (quint., J = 6 Hz, 2H), 3.69 (t, J = 6 Hz, 2H), 4.05 (t, J = 6 Hz, 2H), 6.54 (d, J = 6 Hz, 2H), 6.84 (d, J = 6 Hz, 2H), 7.02 (d, J = 9 Hz, 2H), 7.44 (d, J = 9 Hz, 2H).

^{13}C NMR ($CDCl_3$, 75 MHz): δ (ppm) 25.5, 25.8, 29.1, 32.6, 62.8, 68.2, 114.6, 122.4, 124.6, 131.5, 131.7, 132.3, 135.4, 143.4, 161.7.

HRMS Calc. for $C_{21}H_{21}BF_2Br_2N_2O_2Na^+$ $[M + Na]^+$ 564.9908; Found 564.9880.

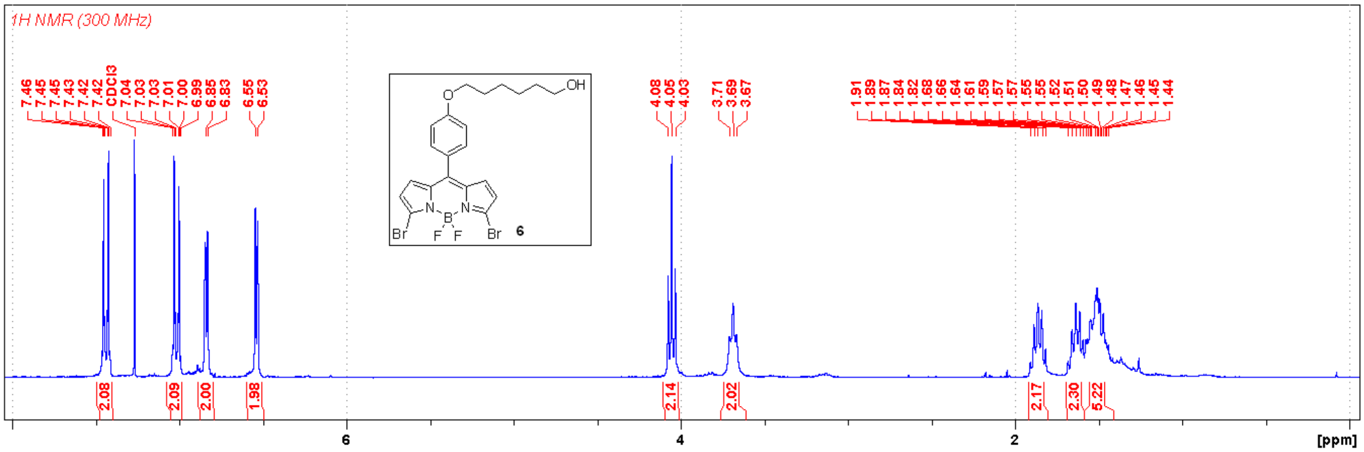

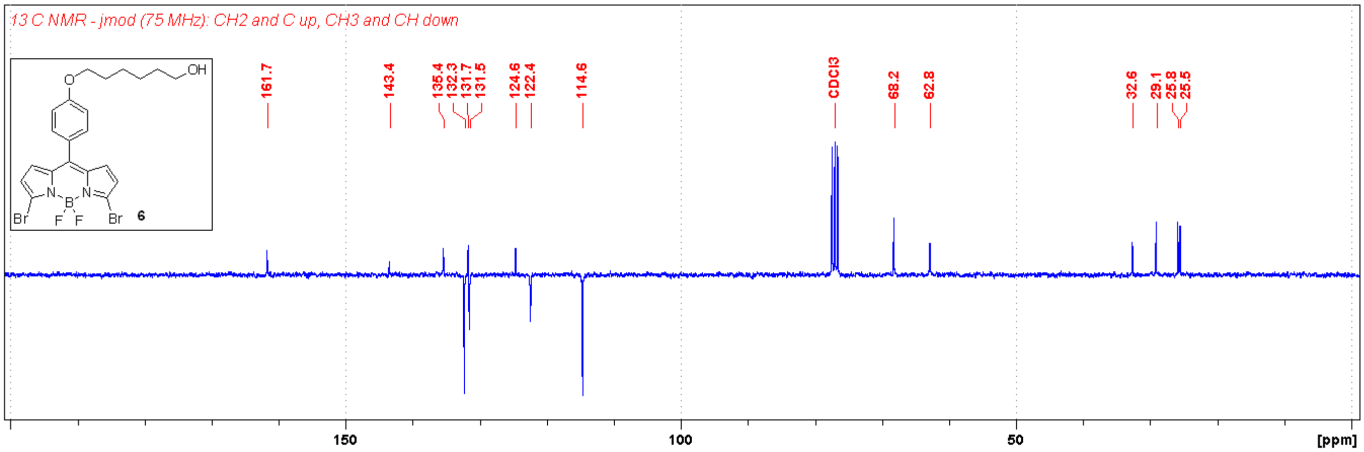

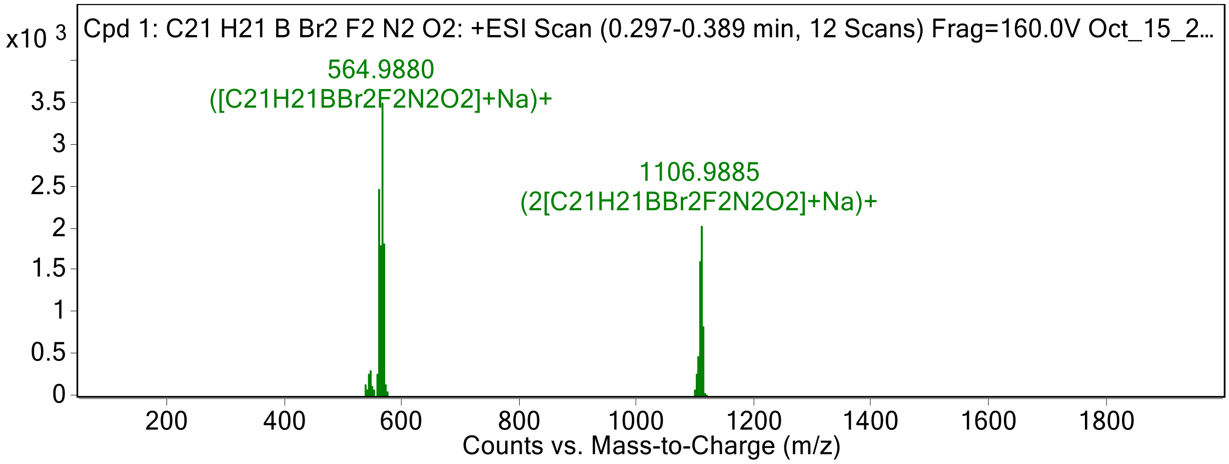

BODIPY-CN alcohol (4).

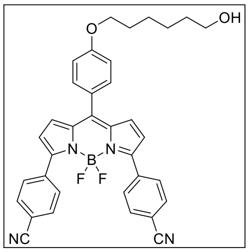

(a) To a round bottomed flask containing aldehyde **1** (500 mg, 2.25 mmol) in dry DCM (10 mL) was added cyanophenyl-substituted pyrrole **2** (832 mg, 4.95 mmol) and catalytic amounts of CF_3COOH (3 drops). The mixture was stirred at room temperature for 4 h under an argon atmosphere. After completion of the reaction, solution was diluted with DCM and washed with water (2X) and brine (1X). The organic layer was then dried over Na_2SO_4 and concentrated to dryness under reduced pressure to obtain crude product **3**, which was dissolved in dry DCM (10 mL) and treated with DDQ (511 mg, 2.25 mmol) dissolved in dry DCM (15 mL) that was added dropwise over 1 h and the reaction mixture was allowed to stir for an additional 1 h. After completion of the reaction, it was in situ treated with $BF_3{\cdot}Et_2O$ (3.5 mL) followed by NEt_3 (0.75 mL) and further stirred for 2 h. After completion, the reaction mixture was filtered, and the filtrate was washed with water (3X) and brine (1X). The organic layer was dried over Na_2SO_4 and concentrated to dryness under reduced pressure. The resulting crude product was purified using a flash column chromatography (silica gel; EtOAc/hexane). The dark orange-red fraction was collected and concentrated to dryness to afford the desired product **4** as a red crystalline solid (211 mg, 16%).

(b) To a stirred solution of BODIPY-dibromide **6** (300 mg, 0.55 mmol) and $Pd(PPh_3)_4$ (32 mg, 5 mol%) in THF−toluene−water (1:1:1, 50 mL), 4-cyanophenyl boronic acid (7, 244 mg, 1.66 mmol) and Na_2CO_3 (235 mg, 2.22 mmol) were added under argon. The mixture was refluxed at 80 °C for 8 h. After completion of reaction, the solution was evaporated under reduced pressure and the residue was purified by column chromatography (silica gel, EtOAc/hexane) to obtain a red solid of **4** (198 mg, 61%).

^{1}H NMR ($CDCl_3$, 300 MHz): δ (ppm) 1.34 (br s, 1H), 1.43−1.72 (m, 6H), 1.90 (quint., J = 6 Hz, 2H), 3.70 (t, J = 6 Hz, 2H), 4.10 (t, J = 6 Hz, 2H), 6.69 (d, J = 6 Hz, 2H), 7.05 (d, J = 6 Hz, 2H) 7.09 (d, J = 9 Hz, 2H), 7.57 (d, J = 9 Hz, 2H), 7.72 (d, J = 6 Hz, 4H), 7.95 (d, J = 6 Hz, 4H).

^{13}C NMR ($CDCl_3$, 75 MHz): δ (ppm) 25.5, 25.8, 29.0, 32.5, 62.7, 68.2, 112.7, 114.5, 118.5, 120.9, 125.9, 129.9, 131.7, 131.9, 132.5, 136.7, 136.8, 146.5, 155.9, 161.7.

HRMS Calc. for $C_{35}H_{29}BF_2N_4O_2Na^+$ [M + Na]$^+$ 609.2249; Found 609.2217.

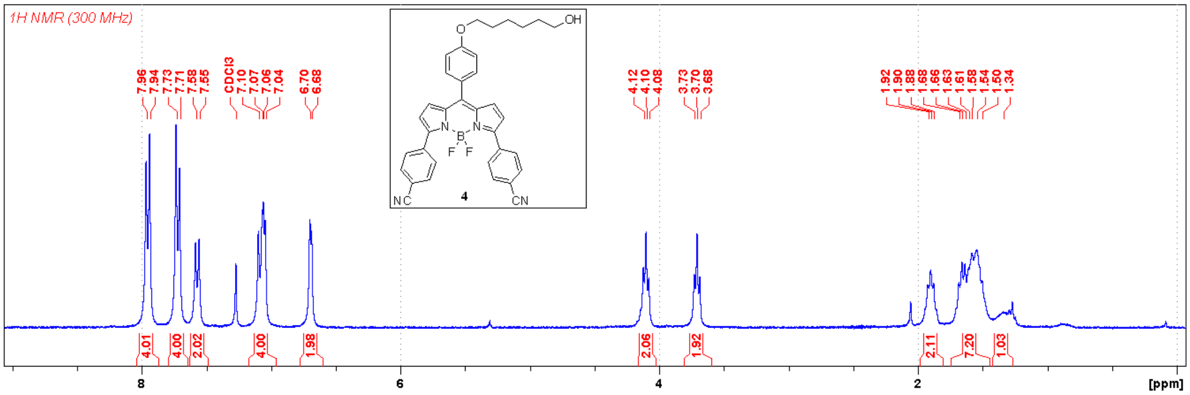

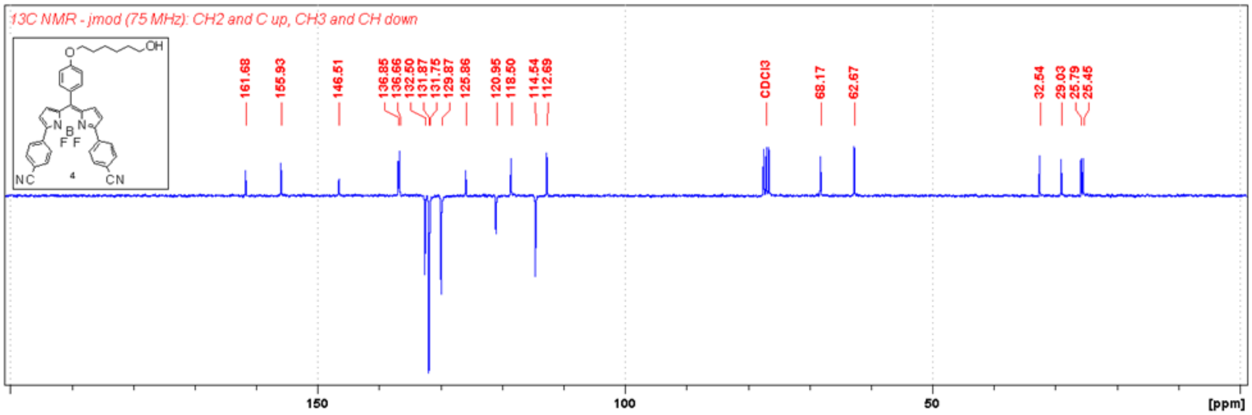

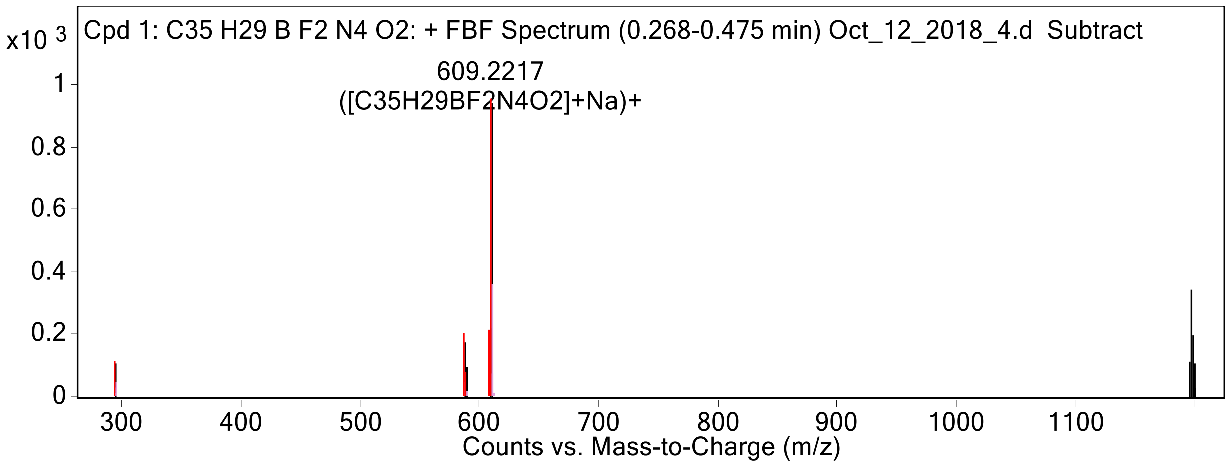

BODIPY-CN phosphoramidite (8).

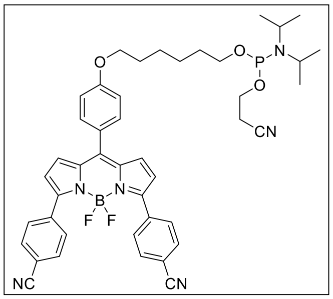

A round bottom flask containing BODIPY-CN alcohol **4** (250 mg, 0.43 mmol) was dried under vacuum and reverse-filled with argon. Dry-degassed DCM (10 mL) was added to it followed by dry-degassed NEt_3 (0.18 mL, 1.29 mmol) and 2-cyanoethyl *N,N*-diisopropylchlorophosphoramidite (0.11 mL, 0.47 mmol). It was then stirred at room temperature for 30 min. After completion of the reaction, the solvent was concentrated to 1/3 volume under reduced pressure. It was then transferred dropwise into dry-degassed hexane to obtain the product precipitate. The precipitate was washed successively with hexane and dried under reduced pressure to obtain BODIPY-CN phosphoramidite **8** (quant.). The phosphoramidite is air sensitive and its purity was checked by NMR (^{1}H and ^{31}P) and then the molecule was immediately used on the DNA synthesizer.

^{1}H NMR ($CDCl_3$, 300 MHz): δ (ppm) 1.20 (d, *J* = 6 Hz, 12H), 1.42−1.58 (m, 4H), 1.69 (quint., *J* = 6 Hz, 2H), 1.88 (quint., *J* = 6 Hz, 2H), 2.65 (t, *J* = 6 Hz, 2H), 3.53−3.72 (m, 4H), 3.73−3.93 (m, 2H), 4.09 (t, *J* = 6 Hz, 2H), 6.69 (d, *J* = 3 Hz, 2H), 7.05 (d, *J* = 3 Hz, 2H), 7.08 (d, *J* = 6 Hz, 2H), 7.57 (d, *J* = 6 Hz, 2H), 7.72 (d, *J* = 6 Hz, 4H), 7.95 (d, *J* = 6 Hz, 4H).

^{31}P NMR ($CDCl_3$, 121 MHz): δ (ppm) 7.8 (9%), 14.3 (8%), 147.5 (83%).

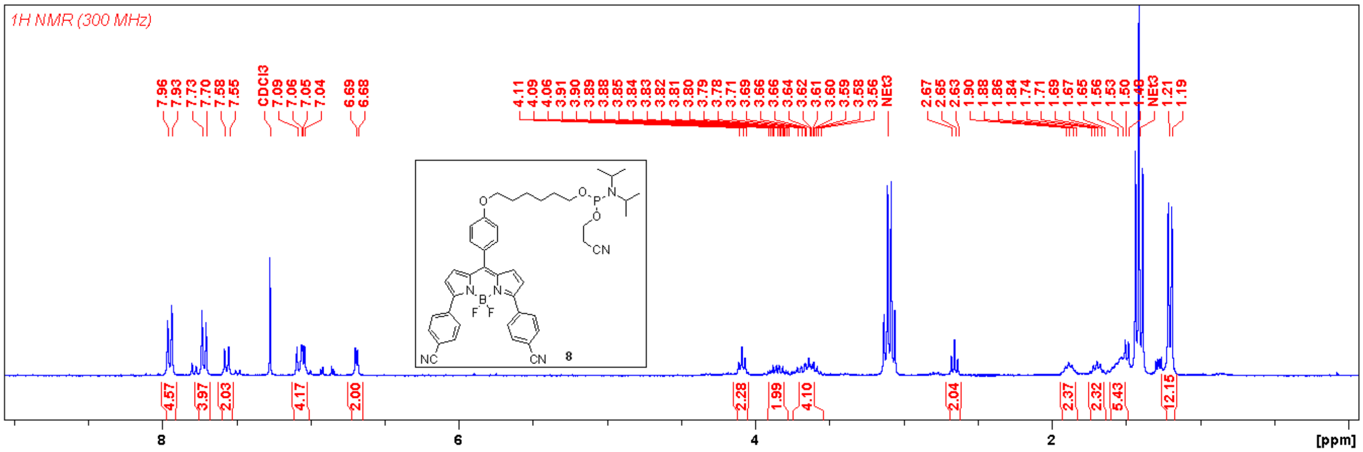

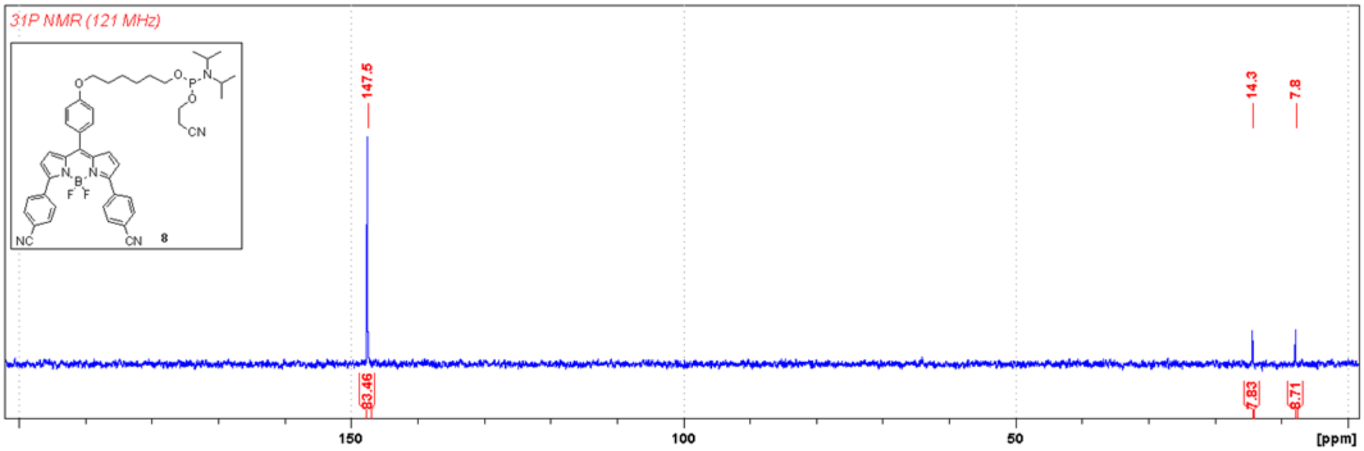

Table S1: Tabulated MS data of BODIPY-CN-TBA.

mTBA	Formula	Calc. Mass	*m/z* (ESI$^-$)	Exp. Mass
BODIPY-CN	$C_{185}H_{215}N_{61}O_{98}P_{15}BF_2$	5372	$[M\text{-}8H]^{8-}$ = 670.5	5372.0
			$[M\text{-}7H]^{7-}$ = 766.6	5373.2
			$[M\text{-}6H]^{6-}$ = 894.6	5373.6
			$[M\text{-}5H]^{5-}$ = 1073.6	5373.0

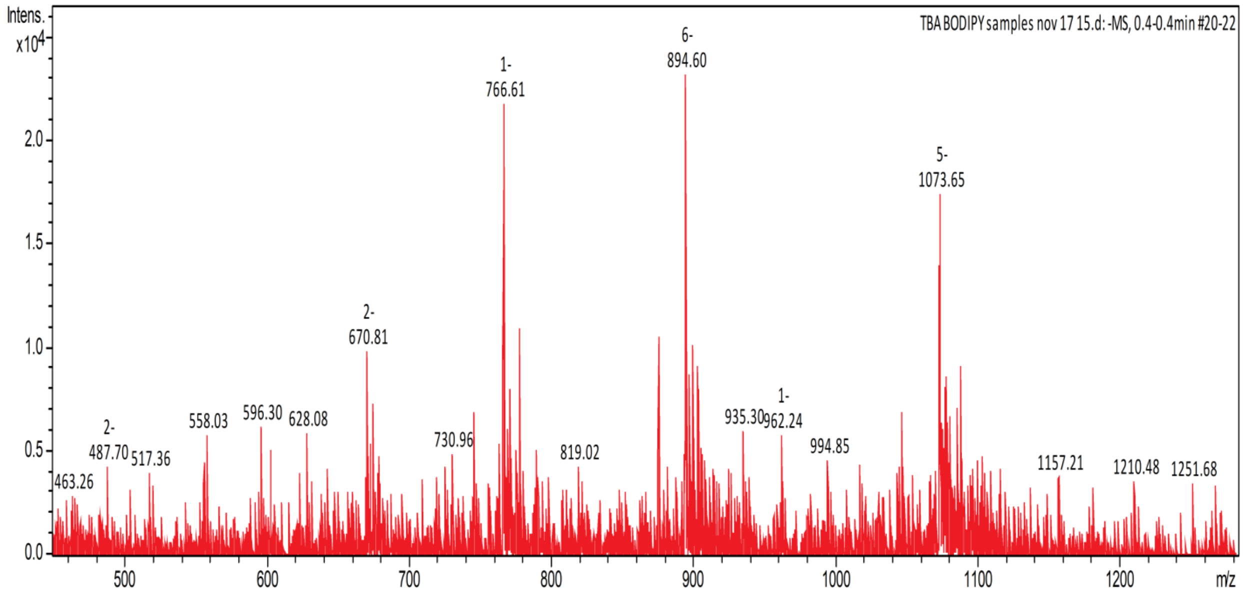

Figure S4 .Negative ESI MS spectrum of BODIPY-CN-TBA.

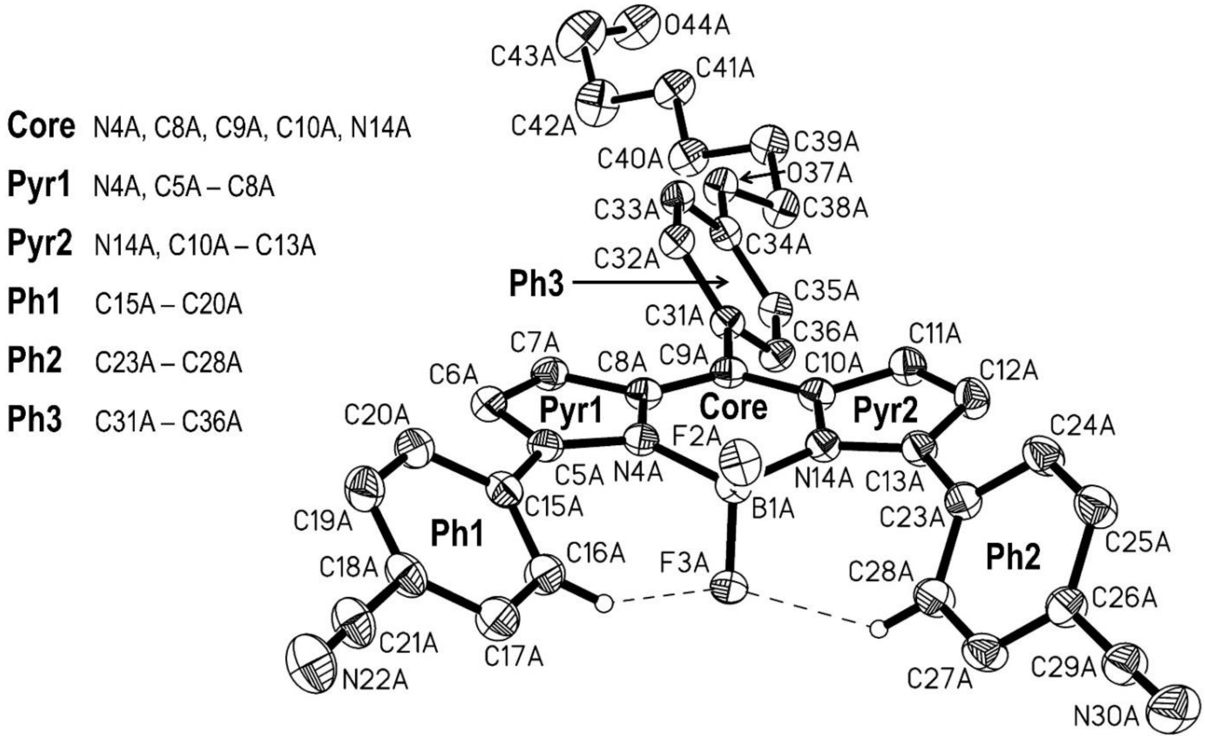

Figure S5. ORTEP drawing of the BODIPY-CN die as found in the crystal structure with labeling scheme for atoms and planes. For clarity, only two H atoms involved in the C-H…F intramolecular H-bonds (dashes) are shown.

Table S2. Least-square planes (see Figure S15 for coding) and dihedral angles between selected planes.

Plane	**Core**	**Pyr1**	**Pyr2**	**Ph1**	**Ph2**	**Ph3**
Max deviation of atoms from the plane, Å	0.029	0.006	0.004	0.007	0.006	0.009
Deviation of B1A atom from the plane, Å	0.192(2)	0.183(2)	0.076(2)			

Planes	**Core - Pyr1**	**Core - Pyr2**	**Pyr1 - Pyr2**	**Pyr1 - Ph1**	**Pyr2 - Ph2**	**Core - Ph3**
Dihedral angle, deg.	3.06(9)	9.54(9)	12.49(9)	33.34(7)	42.89(5)	50.62(4)

Table S3. Crystal data and structure refinement for BODIPY-CN*0.4($CHCl_3$)*0.2(H_2O).

Empirical formula	C35.40 H29.40 B Cl1.19 F2 N4 O2.20	
Formula weight	637.21	
Temperature	150(2) K	
Wavelength	1.54184 Å	
Crystal system	Monoclinic	
Space group	P 2_1/c	
Unit cell dimensions	a = 12.72904(8) Å	α = 90°.
	b = 14.58894(9) Å	β = 100.3226(6)°.
	c = 17.13975(10) Å	γ = 90°.
Volume	3131.39(3) Å^3	
Z	4	
Density (calculated)	1.352 Mg/m^3	
Absorption coefficient	1.667 mm^{-1}	
F(000)	1323	
Crystal size	0.500 x 0.250 x 0.040 mm^3	
Theta range for data collection	3.529 to 75.840°.	
Index ranges	-16<=h<=15, -18<=k<=18, -21<=l<=21	
Reflections collected	76700	
Independent reflections	6508 [R(int) = 0.0350]	
Completeness to theta = 67.684°	99.6 %	
Absorption correction	Semi-empirical from equivalents	
Max. and min. transmission	1.00000 and 0.49925	
Refinement method	Full-matrix least-squares on F^2	
Data / restraints / parameters	6508 / 0 / 528	
Goodness-of-fit on F^2	1.056	
Final R indices [I>2sigma(I)]	R1 = 0.0427, wR2 = 0.1205	
R indices (all data)	R1 = 0.0463, wR2 = 0.1240	

Largest diff. peak and hole	0.539 and -0.260 e.Å^{-3}

Table S4. Atomic coordinates (x 10^4) and equivalent isotropic displacement parameters (Å^{2}x 10^3) for BODIPY-CN*0.4($CHCl_3$)*0.2(H_2O). U(eq) is defined as one third of the trace of the orthogonalized U^{ij} tensor.

	x	y	z	U(eq)
B(1A)	6168(1)	1997(1)	5915(1)	23(1)
C(5A)	4782(1)	655(1)	5927(1)	24(1)
C(6A)	3862(1)	337(1)	5409(1)	27(1)
C(7A)	3644(1)	938(1)	4784(1)	26(1)
C(8A)	4423(1)	1640(1)	4923(1)	23(1)
C(9A)	4520(1)	2443(1)	4495(1)	23(1)
C(10A)	5333(1)	3079(1)	4766(1)	24(1)
C(11A)	5475(1)	3980(1)	4512(1)	29(1)
C(12A)	6325(1)	4351(1)	5030(1)	30(1)
C(13A)	6707(1)	3674(1)	5593(1)	25(1)
C(15A)	5265(1)	189(1)	6666(1)	25(1)
C(16A)	6362(1)	159(1)	6958(1)	30(1)
C(17A)	6763(1)	-329(1)	7640(1)	33(1)
C(18A)	6065(1)	-794(1)	8045(1)	30(1)
C(19A)	4973(1)	-782(1)	7757(1)	34(1)
C(20A)	4580(1)	-297(1)	7073(1)	32(1)
C(21A)	6492(1)	-1296(1)	8754(1)	34(1)
C(23A)	7640(1)	3782(1)	6231(1)	25(1)
C(24A)	7785(1)	4616(1)	6637(1)	33(1)
C(25A)	8688(1)	4772(1)	7206(1)	35(1)
C(26A)	9468(1)	4095(1)	7367(1)	29(1)
C(27A)	9341(1)	3263(1)	6964(1)	33(1)
C(28A)	8426(1)	3106(1)	6405(1)	30(1)
C(29A)	10412(1)	4274(1)	7954(1)	33(1)
C(31A)	3735(1)	2654(1)	3772(1)	24(1)
C(32A)	2637(1)	2622(1)	3785(1)	25(1)
C(33A)	1901(1)	2833(1)	3115(1)	25(1)
C(34A)	2240(1)	3067(1)	2410(1)	24(1)
C(35A)	3328(1)	3112(1)	2387(1)	26(1)
C(36A)	4066(1)	2908(1)	3069(1)	26(1)
C(38A)	1749(1)	3693(1)	1098(1)	32(1)
C(39A)	736(1)	3929(1)	523(1)	35(1)
C(40A)	169(1)	3099(1)	109(1)	36(1)

C(41A)	-917(1)	3332(1)	-393(1)	38(1)
C(42A)	-1472(2)	2501(1)	-816(1)	50(1)
C(43A)	-2577(2)	2710(2)	-1282(1)	61(1)
N(4A)	5123(1)	1445(1)	5630(1)	22(1)
N(14A)	6099(1)	2904(1)	5435(1)	22(1)
N(22A)	6847(1)	-1690(1)	9318(1)	43(1)
N(30A)	11149(1)	4428(1)	8423(1)	44(1)
O(37A)	1444(1)	3258(1)	1782(1)	28(1)
O(44A)	-2603(2)	3305(2)	-1859(1)	51(1)
O(44B)	-3006(5)	1972(4)	-1670(3)	110(2)
F(2A)	6284(1)	2195(1)	6718(1)	30(1)
F(3A)	7031(1)	1482(1)	5761(1)	28(1)
C(1C)	10029(4)	-282(4)	9803(3)	53(1)
Cl(2C)	9092(1)	138(1)	9015(1)	83(1)
Cl(3C)	11020(1)	520(1)	10107(1)	74(1)
Cl(4C)	9347(2)	-601(1)	10566(1)	84(1)
O(1D)	9200(10)	-446(8)	9424(8)	93(3)

Table S5. Bond lengths [Å] and angles [°] for BODIPY-CN*0.4($CHCl_3$)*0.2(H_2O).

B(1A)-F(2A)	1.3885(15)
B(1A)-F(3A)	1.3950(15)
B(1A)-N(14A)	1.5526(16)
B(1A)-N(4A)	1.5569(16)
C(5A)-N(4A)	1.3624(16)
C(5A)-C(6A)	1.4140(18)
C(5A)-C(15A)	1.4711(17)
C(6A)-C(7A)	1.3739(19)
C(6A)-H(31)	0.936(18)
C(7A)-C(8A)	1.4163(17)
C(7A)-H(24)	0.959(17)
C(8A)-C(9A)	1.3987(18)
C(8A)-N(4A)	1.3993(15)
C(9A)-C(10A)	1.4055(17)
C(9A)-C(31A)	1.4788(15)
C(10A)-N(14A)	1.3892(15)
C(10A)-C(11A)	1.4071(18)
C(11A)-C(12A)	1.3808(18)
C(11A)-H(19)	0.991(18)
C(12A)-C(13A)	1.4052(17)
C(12A)-H(36)	0.941(19)
C(13A)-N(14A)	1.3629(16)

C(13A)-C(23A)	1.4720(16)
C(15A)-C(16A)	1.3974(19)
C(15A)-C(20A)	1.4026(18)
C(16A)-C(17A)	1.3859(18)
C(16A)-H(37)	0.946(19)
C(17A)-C(18A)	1.396(2)
C(17A)-H(34)	0.96(2)
C(18A)-C(19A)	1.389(2)
C(18A)-C(21A)	1.4397(18)
C(19A)-C(20A)	1.3845(19)
C(19A)-H(12)	0.97(2)
C(20A)-H(32)	0.981(19)
C(21A)-N(22A)	1.145(2)
C(23A)-C(28A)	1.3977(18)
C(23A)-C(24A)	1.3980(18)
C(24A)-C(25A)	1.3863(19)
C(24A)-H(6)	0.95(2)
C(25A)-C(26A)	1.3937(19)
C(25A)-H(4)	0.97(2)
C(26A)-C(27A)	1.3914(19)
C(26A)-C(29A)	1.4464(17)
C(27A)-C(28A)	1.3881(18)
C(27A)-H(28)	0.970(19)
C(28A)-H(0AA)	0.985(19)
C(29A)-N(30A)	1.1430(19)
C(31A)-C(36A)	1.3956(18)
C(31A)-C(32A)	1.4026(17)
C(32A)-C(33A)	1.3808(17)
C(32A)-H(27)	0.966(17)
C(33A)-C(34A)	1.3972(18)
C(33A)-H(29)	1.005(17)
C(34A)-O(37A)	1.3672(14)
C(34A)-C(35A)	1.3940(18)
C(35A)-C(36A)	1.3942(17)
C(35A)-H(17)	0.958(18)
C(36A)-H(16)	0.976(18)
C(38A)-O(37A)	1.4473(16)
C(38A)-C(39A)	1.5146(18)
C(38A)-H(1AA)	0.948(19)
C(38A)-H(1AB)	1.025(19)
C(39A)-C(40A)	1.518(2)
C(39A)-H(2AA)	1.00(2)
C(39A)-H(2AB)	1.00(2)
C(40A)-C(41A)	1.5297(19)
C(40A)-H(3AA)	1.00(2)

C(40A)-H(3AB)	1.01(2)
C(41A)-C(42A)	1.520(2)
C(41A)-H(7A)	1.04(2)
C(41A)-H(7B)	1.04(2)
C(42A)-C(43A)	1.519(2)
C(42A)-H(5AA)	1.05(2)
C(42A)-H(5AB)	1.05(2)
C(43A)-O(44A)	1.311(3)
C(43A)-O(44B)	1.330(5)
C(43A)-H(6AC)	0.9900
C(43A)-H(6AD)	0.9900
C(43A)-H(6AA)	0.9900
C(43A)-H(6AB)	0.9900
O(44A)-H(1)	0.8400
O(44B)-H(0AB)	0.8400
C(1C)-Cl(3C)	1.731(5)
C(1C)-Cl(2C)	1.745(5)
C(1C)-Cl(4C)	1.756(6)
C(1C)-H(3)	1.0000

F(2A)-B(1A)-F(3A)	110.69(10)
F(2A)-B(1A)-N(14A)	109.48(10)
F(3A)-B(1A)-N(14A)	109.28(10)
F(2A)-B(1A)-N(4A)	110.57(10)
F(3A)-B(1A)-N(4A)	108.64(10)
N(14A)-B(1A)-N(4A)	108.13(9)
N(4A)-C(5A)-C(6A)	109.03(11)
N(4A)-C(5A)-C(15A)	126.89(11)
C(6A)-C(5A)-C(15A)	124.08(12)
C(7A)-C(6A)-C(5A)	108.04(11)
C(7A)-C(6A)-H(31)	127.4(11)
C(5A)-C(6A)-H(31)	124.5(11)
C(6A)-C(7A)-C(8A)	107.04(11)
C(6A)-C(7A)-H(24)	128.1(10)
C(8A)-C(7A)-H(24)	124.9(10)
C(9A)-C(8A)-N(4A)	121.55(11)
C(9A)-C(8A)-C(7A)	129.96(11)
N(4A)-C(8A)-C(7A)	108.41(11)
C(8A)-C(9A)-C(10A)	120.50(11)
C(8A)-C(9A)-C(31A)	120.31(11)
C(10A)-C(9A)-C(31A)	119.12(11)
N(14A)-C(10A)-C(9A)	121.24(11)
N(14A)-C(10A)-C(11A)	108.47(11)
C(9A)-C(10A)-C(11A)	129.94(11)
C(12A)-C(11A)-C(10A)	107.23(11)

C(12A)-C(11A)-H(19)	127.6(10)
C(10A)-C(11A)-H(19)	125.2(10)
C(11A)-C(12A)-C(13A)	107.53(12)
C(11A)-C(12A)-H(36)	128.0(11)
C(13A)-C(12A)-H(36)	124.4(11)
N(14A)-C(13A)-C(12A)	109.17(11)
N(14A)-C(13A)-C(23A)	126.07(11)
C(12A)-C(13A)-C(23A)	124.72(12)
C(16A)-C(15A)-C(20A)	118.48(12)
C(16A)-C(15A)-C(5A)	124.04(12)
C(20A)-C(15A)-C(5A)	117.38(12)
C(17A)-C(16A)-C(15A)	120.73(13)
C(17A)-C(16A)-H(37)	120.0(11)
C(15A)-C(16A)-H(37)	119.3(11)
C(16A)-C(17A)-C(18A)	119.88(13)
C(16A)-C(17A)-H(34)	120.5(11)
C(18A)-C(17A)-H(34)	119.6(11)
C(19A)-C(18A)-C(17A)	120.20(12)
C(19A)-C(18A)-C(21A)	120.50(13)
C(17A)-C(18A)-C(21A)	119.30(13)
C(20A)-C(19A)-C(18A)	119.57(13)
C(20A)-C(19A)-H(12)	122.4(11)
C(18A)-C(19A)-H(12)	118.1(11)
C(19A)-C(20A)-C(15A)	121.12(13)
C(19A)-C(20A)-H(32)	117.6(11)
C(15A)-C(20A)-H(32)	121.2(11)
N(22A)-C(21A)-C(18A)	178.88(18)
C(28A)-C(23A)-C(24A)	118.88(11)
C(28A)-C(23A)-C(13A)	122.32(11)
C(24A)-C(23A)-C(13A)	118.62(11)
C(25A)-C(24A)-C(23A)	120.75(12)
C(25A)-C(24A)-H(6)	119.9(11)
C(23A)-C(24A)-H(6)	119.3(11)
C(24A)-C(25A)-C(26A)	119.66(13)
C(24A)-C(25A)-H(4)	120.9(11)
C(26A)-C(25A)-H(4)	119.5(11)
C(27A)-C(26A)-C(25A)	120.31(12)
C(27A)-C(26A)-C(29A)	120.76(12)
C(25A)-C(26A)-C(29A)	118.94(12)
C(28A)-C(27A)-C(26A)	119.68(12)
C(28A)-C(27A)-H(28)	118.6(11)
C(26A)-C(27A)-H(28)	121.7(11)
C(27A)-C(28A)-C(23A)	120.71(12)
C(27A)-C(28A)-H(0AA)	119.8(11)
C(23A)-C(28A)-H(0AA)	119.5(11)

N(30A)-C(29A)-C(26A)	178.81(16)
C(36A)-C(31A)-C(32A)	118.63(11)
C(36A)-C(31A)-C(9A)	121.04(11)
C(32A)-C(31A)-C(9A)	120.31(11)
C(33A)-C(32A)-C(31A)	120.58(11)
C(33A)-C(32A)-H(27)	121.9(10)
C(31A)-C(32A)-H(27)	117.5(10)
C(32A)-C(33A)-C(34A)	120.33(11)
C(32A)-C(33A)-H(29)	119.9(9)
C(34A)-C(33A)-H(29)	119.7(9)
O(37A)-C(34A)-C(35A)	124.61(11)
O(37A)-C(34A)-C(33A)	115.48(11)
C(35A)-C(34A)-C(33A)	119.89(11)
C(34A)-C(35A)-C(36A)	119.37(11)
C(34A)-C(35A)-H(17)	120.6(10)
C(36A)-C(35A)-H(17)	120.1(10)
C(35A)-C(36A)-C(31A)	121.17(11)
C(35A)-C(36A)-H(16)	119.0(10)
C(31A)-C(36A)-H(16)	119.8(10)
O(37A)-C(38A)-C(39A)	107.83(11)
O(37A)-C(38A)-H(1AA)	109.7(11)
C(39A)-C(38A)-H(1AA)	111.2(11)
O(37A)-C(38A)-H(1AB)	107.9(10)
C(39A)-C(38A)-H(1AB)	112.8(10)
H(1AA)-C(38A)-H(1AB)	107.4(15)
C(38A)-C(39A)-C(40A)	113.66(13)
C(38A)-C(39A)-H(2AA)	110.5(11)
C(40A)-C(39A)-H(2AA)	109.0(11)
C(38A)-C(39A)-H(2AB)	108.6(11)
C(40A)-C(39A)-H(2AB)	106.7(11)
H(2AA)-C(39A)-H(2AB)	108.2(16)
C(39A)-C(40A)-C(41A)	113.07(13)
C(39A)-C(40A)-H(3AA)	108.6(11)
C(41A)-C(40A)-H(3AA)	109.5(11)
C(39A)-C(40A)-H(3AB)	109.6(11)
C(41A)-C(40A)-H(3AB)	109.6(11)
H(3AA)-C(40A)-H(3AB)	106.3(16)
C(42A)-C(41A)-C(40A)	112.78(14)
C(42A)-C(41A)-H(7A)	110.8(11)
C(40A)-C(41A)-H(7A)	107.6(11)
C(42A)-C(41A)-H(7B)	108.7(11)
C(40A)-C(41A)-H(7B)	110.3(11)
H(7A)-C(41A)-H(7B)	106.6(15)
C(43A)-C(42A)-C(41A)	113.48(17)
C(43A)-C(42A)-H(5AA)	111.8(13)

C(41A)-C(42A)-H(5AA)	110.3(12)
C(43A)-C(42A)-H(5AB)	108.9(12)
C(41A)-C(42A)-H(5AB)	110.3(13)
H(5AA)-C(42A)-H(5AB)	101.4(17)
O(44A)-C(43A)-C(42A)	115.20(19)
O(44B)-C(43A)-C(42A)	111.1(4)
O(44A)-C(43A)-H(6AC)	108.5
C(42A)-C(43A)-H(6AC)	108.5
O(44A)-C(43A)-H(6AD)	108.5
C(42A)-C(43A)-H(6AD)	108.5
H(6AC)-C(43A)-H(6AD)	107.5
O(44B)-C(43A)-H(6AA)	109.4
C(42A)-C(43A)-H(6AA)	109.4
O(44B)-C(43A)-H(6AB)	109.4
C(42A)-C(43A)-H(6AB)	109.4
H(6AA)-C(43A)-H(6AB)	108.0
C(5A)-N(4A)-C(8A)	107.47(10)
C(5A)-N(4A)-B(1A)	129.25(10)
C(8A)-N(4A)-B(1A)	122.78(10)
C(13A)-N(14A)-C(10A)	107.59(10)
C(13A)-N(14A)-B(1A)	128.41(10)
C(10A)-N(14A)-B(1A)	123.93(10)
C(34A)-O(37A)-C(38A)	117.30(10)
C(43A)-O(44A)-H(1)	109.5
C(43A)-O(44B)-H(0AB)	109.5
Cl(3C)-C(1C)-Cl(2C)	110.8(3)
Cl(3C)-C(1C)-Cl(4C)	112.7(3)
Cl(2C)-C(1C)-Cl(4C)	108.0(3)
Cl(3C)-C(1C)-H(3)	108.4
Cl(2C)-C(1C)-H(3)	108.4
Cl(4C)-C(1C)-H(3)	108.4

Table S6. Anisotropic displacement parameters ($Å^2$x 10^3) for BODIPY-CN*0.4($CHCl_3$)*0.2(H_2O). The anisotropic displacement factor exponent takes the form: $-2\pi^2$[$h^2a^{*2}U^{11}$ + ... + 2 h k a* b* U^{12}]

	U^{11}	U^{22}	U^{33}	U^{23}	U^{13}	U^{12}
B(1A)	22(1)	24(1)	20(1)	1(1)	-1(1)	2(1)
C(5A)	23(1)	24(1)	26(1)	1(1)	6(1)	3(1)
C(6A)	25(1)	26(1)	31(1)	-1(1)	6(1)	0(1)

C(7A)	22(1)	28(1)	26(1)	-3(1)	2(1)	1(1)
C(8A)	21(1)	26(1)	20(1)	-2(1)	1(1)	2(1)
C(9A)	21(1)	27(1)	20(1)	-1(1)	1(1)	3(1)
C(10A)	22(1)	27(1)	20(1)	1(1)	-1(1)	3(1)
C(11A)	28(1)	28(1)	28(1)	5(1)	-3(1)	2(1)
C(12A)	31(1)	25(1)	33(1)	5(1)	-4(1)	-1(1)
C(13A)	24(1)	24(1)	25(1)	0(1)	0(1)	1(1)
C(15A)	30(1)	22(1)	25(1)	2(1)	6(1)	3(1)
C(16A)	29(1)	32(1)	29(1)	7(1)	5(1)	2(1)
C(17A)	32(1)	36(1)	31(1)	9(1)	2(1)	2(1)
C(18A)	43(1)	24(1)	23(1)	3(1)	6(1)	4(1)
C(19A)	39(1)	30(1)	33(1)	8(1)	12(1)	0(1)
C(20A)	30(1)	30(1)	35(1)	7(1)	7(1)	2(1)
C(21A)	48(1)	28(1)	26(1)	2(1)	8(1)	0(1)
C(23A)	24(1)	25(1)	24(1)	1(1)	-1(1)	0(1)
C(24A)	30(1)	26(1)	40(1)	-4(1)	-7(1)	4(1)
C(25A)	33(1)	28(1)	40(1)	-8(1)	-7(1)	2(1)
C(26A)	25(1)	31(1)	28(1)	-2(1)	-4(1)	0(1)
C(27A)	28(1)	31(1)	35(1)	-4(1)	-5(1)	6(1)
C(28A)	29(1)	27(1)	32(1)	-5(1)	-3(1)	2(1)
C(29A)	28(1)	34(1)	33(1)	-5(1)	-2(1)	1(1)
C(31A)	23(1)	25(1)	21(1)	0(1)	-1(1)	1(1)
C(32A)	24(1)	28(1)	21(1)	0(1)	1(1)	0(1)
C(33A)	22(1)	28(1)	24(1)	-1(1)	-1(1)	-1(1)
C(34A)	25(1)	22(1)	21(1)	0(1)	-4(1)	-1(1)
C(35A)	27(1)	28(1)	21(1)	2(1)	2(1)	-1(1)
C(36A)	22(1)	31(1)	24(1)	1(1)	1(1)	0(1)
C(38A)	32(1)	38(1)	23(1)	7(1)	-2(1)	-2(1)
C(39A)	35(1)	40(1)	27(1)	9(1)	-4(1)	1(1)
C(40A)	33(1)	45(1)	28(1)	2(1)	-2(1)	1(1)
C(41A)	31(1)	53(1)	28(1)	4(1)	-2(1)	0(1)
C(42A)	48(1)	60(1)	36(1)	0(1)	-7(1)	-6(1)
C(43A)	46(1)	88(2)	43(1)	-2(1)	-10(1)	-11(1)
N(4A)	21(1)	23(1)	20(1)	1(1)	2(1)	2(1)
N(14A)	21(1)	23(1)	20(1)	0(1)	-1(1)	1(1)
N(22A)	59(1)	40(1)	30(1)	10(1)	5(1)	1(1)
N(30A)	31(1)	51(1)	43(1)	-10(1)	-7(1)	0(1)
O(37A)	25(1)	34(1)	21(1)	5(1)	-5(1)	-3(1)
O(44A)	44(1)	75(2)	32(1)	4(1)	-4(1)	-1(1)
O(44B)	128(4)	129(4)	58(2)	13(2)	-25(2)	-76(4)
F(2A)	38(1)	32(1)	19(1)	0(1)	-1(1)	-2(1)
F(3A)	23(1)	25(1)	34(1)	4(1)	3(1)	4(1)
C(1C)	43(2)	59(3)	56(3)	-21(2)	7(2)	11(2)
Cl(2C)	74(1)	94(1)	71(1)	-17(1)	-11(1)	4(1)
Cl(3C)	44(1)	83(1)	92(1)	-30(1)	2(1)	-12(1)

Cl(4C)	86(1)	84(1)	91(1)	4(1)	43(1)	15(1)

Table S7. Hydrogen coordinates (x 10^4) and isotropic displacement parameters ($Å^2$x 10^3) for BODIPY-CN*0.4($CHCl_3$)*0.2(H_2O).

	x	y	z	U(eq)
H(31)	3498(14)	-204(13)	5482(10)	33
H(24)	3079(14)	907(12)	4333(10)	31
H(19)	5040(14)	4277(12)	4042(10)	35
H(36)	6634(15)	4934(13)	5010(10)	37
H(37)	6834(15)	471(13)	6682(10)	36
H(34)	7518(16)	-345(13)	7842(11)	40
H(12)	4513(15)	-1125(13)	8045(11)	40
H(32)	3803(16)	-284(13)	6895(11)	38
H(6)	7263(15)	5083(13)	6513(11)	40
H(4)	8785(15)	5350(14)	7495(11)	42
H(28)	9883(15)	2788(13)	7056(11)	39
H(0AA)	8332(14)	2515(13)	6123(11)	36
H(27)	2423(14)	2466(12)	4282(10)	30
H(29)	1118(14)	2836(11)	3138(10)	30
H(17)	3569(13)	3278(12)	1908(10)	31
H(16)	4825(14)	2907(12)	3041(10)	31
H(1AA)	2188(15)	3291(13)	863(11)	38
H(1AB)	2198(15)	4259(13)	1291(11)	38
H(2AA)	892(15)	4371(13)	114(11)	42
H(2AB)	225(16)	4225(13)	825(11)	42
H(3AA)	72(16)	2637(14)	518(12)	43
H(3AB)	637(16)	2801(14)	-235(11)	43
H(7A)	-1382(16)	3626(14)	-20(12)	46
H(7B)	-829(16)	3826(14)	-814(12)	46
H(5AA)	-1488(18)	1963(16)	-413(13)	59
H(5AB)	-1005(19)	2217(16)	-1200(14)	59
H(6AC)	-2901	2130	-1509	73
H(6AD)	-3026	2945	-911	73
H(6AA)	-3043	2924	-915	73
H(6AB)	-2526	3208	-1666	73
H(1)	-2747	3032	-2297	77
H(0AB)	-2912	1516	-1367	166
H(3)	10367	-842	9620	64
